# Usefulness of plasma bile acid profile as a prognostic biomarker for drug-induced liver injury

**DOI:** 10.1016/j.ebiom.2026.106229

**Published:** 2026-03-24

**Authors:** Maria J. Monte, Thi Dong-Binh Tran, Jane I. Grove, Dingzhou Li, Camilla Stephens, M. Isabel Lucena, Raúl J. Andrade, Sabine Weber, Alexander Gerbes, Einar S. Bjornsson, Guido Stirnimann, Helgi K. Bjornsson, Ann K. Daly, Anthony Evans, Shashi K. Ramaiah, Sara A. Paciga, Melanie Lingaya, Edmond Atallah, Mercedes Robles-Diaz, Sophia L. Samodelov, Oliver Poetz, William Rosenberg, John Ramage, Andrew Fowell, William J.H. Griffiths, Matthew E. Cramp, Janisha Patel, Ahmed M. Elsharkawy, Jose J.G. Marin, Gerd A. Kullak-Ublick, Guruprasad P. Aithal

**Affiliations:** a Experimental Hepatology and Drug Targeting (HEVEPHARM), Institute of Biomedical Research of Salamanca (IBSAL), University of Salamanca, Salamanca, Spain; b Centro de Investigación Biomédica en Red de Enfermedades Hepáticas y Digestivas (CIBERehd), Madrid, Spain; c Data Sciences and Analytics, Pfizer, Groton, Connecticut, USA; d Nottingham Digestive Diseases Centre, Translational Medical Sciences, School of Medicine, University of Nottingham, Nottingham, United Kingdom; e NIHR Nottingham Biomedical Research Centre, Nottingham University Hospitals NHS Trust and the University of Nottingham, Nottingham, United Kingdom; f Servicios de Aparato Digestivo y Farmacologia Clínica, Instituto de Investigación Biomédica de Málaga-IBIMA Plataforma Bionand, Hospital Universitario Virgen de la Victoria, Universidad de Málaga, Malaga, Spain; g Department of Medicine II, Liver Centre Munich, University Hospital, LMU Munich, Germany; h Department of Gastroenterology, Landspitali University Hospital Reykjavik, University of Iceland, Reykjavík, Iceland; i Faculty of Medicine, University of Iceland, Reykjavík, Iceland; j University Clinic for Visceral Surgery and Medicine, University Hospital Inselspital and University of Bern, Bern, Switzerland; k Sahlgrenska University Hospital, Department of Internal Medicine, Division of Gastroenterology and Hepatology, Gothenburg, Sweden; l Translational and Clinical Research Institute, Newcastle University, Newcastle upon Tyne, United Kingdom; m Computational Biology Facility, University of Liverpool, Liverpool, United Kingdom; n Drug Safety Research and Development, Pfizer Inc., Cambridge, Massachusetts, USA; o Department of Clinical Pharmacology and Toxicology, University Hospital Zurich, University of Zurich, 8006 Zurich, Switzerland; p Signatope GmbH, 72770, Reutlingen, Germany; q Royal Free Hospital, London, United Kingdom; r Hampshire Hospitals NHS Foundation Trust, Basingstoke, United Kingdom; s Queen Alexandra Hospital, Portsmouth, United Kingdom; t Department of Hepatology, Addenbrookes University Hospital, Cambridge, United Kingdom; u South West Liver Unit, University Hospitals Plymouth NHS Trust, Plymouth, United Kingdom; v University Hospital Southampton NHS Foundation Trust, Southampton, United Kingdom; w Liver Unit and NIHR Biomedical Research Unit at University Hospitals Birmingham, Birmingham, United Kingdom; x Mechanistic Safety, Patient Safety and Pharmacovigilance, Novartis Pharma, 4056 Basel, Switzerland

**Keywords:** Hepatotoxicity, Cholestasis, Model for end-stage liver disease (MELD), Adverse drug reaction

## Abstract

**Background:**

The liver maintains bile acid (BA) homoeostasis; circulating BA levels are used as a biomarker in certain cholestatic conditions. BAs can initiate processes in the pathogenesis of drug-induced liver injury (DILI), an unpredictable occurrence which can lead to liver failure. As such, this study aimed to explore whether changes in plasma BA profiles can serve as useful biomarkers for diagnostic and prognostic purposes in patients presenting with suspected DILI.

**Methods:**

In a prospective, nested case–control observational study, patients presenting with acute liver injury potentially due to DILI were sampled and followed through standard clinical care with severity and outcomes monitored. After review, cases were adjudicated as DILI or nonDILI (alternate causes). Plasma BA levels and profile were quantified and compared to those in healthy volunteers (n = 25).

**Findings:**

Total plasma BA levels in patients with DILI (n = 120) were significantly elevated compared to healthy volunteers; the nonDILI group (n = 49) also displayed marked hypercholanemia. Higher values of total, primary, and conjugated BAs at presentation, were associated with liver injury that was likely to progress in severity. The ratios of primary-to-secondary BAs and (cholic acid + deoxycholic acid) to (chenodeoxycholic acid + lithocholic acid) improved the prognostic value of the model for end-stage liver disease (MELD) score.

**Interpretation:**

BA profiling could be useful for the early detection of patients where DILI is likely to become more severe and those with outcomes of death or liver transplantation. Further investigation in another independent longitudinal study is needed to validate this biomarker.

**Funding:**

IMI2 821283; IS-BRC-1215-20003.

**Research in context**

**Evidence before this study**

Within the liver, transporting polypeptides are involved in mediating bile acid (BA) uptake, (i.e. BA recovery from sinusoidal blood), and uptake of a variety of compounds and drugs. During drug-induced liver injury (DILI), hepatotoxicity disrupts normal functioning in BA homoeostasis. Further pathway analysis has revealed intrahepatic BAs contribute to pathways connected to disease mechanisms driving DILI.

Disruption of BA homoeostasis reflecting perturbations in metabolism and liver function can indicate cholestatic disorders. Although available in many hospitals, measurement of total BA concentrations is currently usually only used for pregnancy-induced intrahepatic cholestasis and genetic cholestasis.

A recent systematic review including 1630 patients and 836 controls published between 1990 and 2017 concluded that there was a lack of solid evidence to support the use of individual BAs or BA ratios as biomarkers of liver injury with the exception of intrahepatic cholestasis of pregnancy. We also carried out a systematic review for 14 studies which highlighted a case of need for non-genetic biomarkers with the potential to identify serious adverse outcomes from acute DILI.

A search for relevant studies in PubMed published up to 1st Jan 2019 (when this study was initiated) using “bile acid” AND (“drug-induced liver injury” OR “DILI”) AND “biomarker”, without language restrictions, found 8 studies reporting observational studies assessing BAs as a biomarker of liver function relating to DILI. In 5 animal studies and an in vitro investigation, the potential utility of BAs as a non-invasive marker of impaired hepatobiliary transport or injury due to specific drugs (methapyrilene, troglitazone) is described. In human studies, glycodeoxycholic acid levels are reported as prognostic biomarker in acetaminophen-induced acute liver failure and total serum BAs were shown to be elevated in workers exposed to compounds in organic solvents. Previous studies have not compared bile acid profiles in DILI with those in other types of acute liver injury meeting the same diagnostic criteria.

**Added value of this study**

We designed a study to include all suspected DILI cases presenting in a clinical setting to establish a large prospective, multi-national cohort with robust causality assessment and panel adjudication so that BA profiling could be evaluated as a diagnostic and prognostic biomarker.

Total plasma BA levels in patients with DILI were significantly elevated compared to levels in healthy volunteers. Higher values of total, primary, and conjugated BAs at presentation, were associated with liver injury that was likely to progress in severity. BA ratios at the time of acute liver injury were found to improve the performance of MELD score in identifying patients who may require liver transplantation or at increased risk of death. Levels of total BAs were also increased in patients who presented with raised liver enzymes and suspicion of DILI who were later diagnosed with alternate disorders after investigation (nonDILI). BA profiles were not able to distinguish DILI from nonDILI cases.

**Implications of all the available evidence**

Determination of plasma concentrations of total, individual BAs and their ratios can be used for identification of patients with DILI who are likely to progress, require transplantation or die. As methods for measuring plasma BAs are widely accessible, this could be used for the evaluation of patients with DILI.

## Introduction

Bile acids (BAs), the main organic constituent of bile, play a crucial role, not only in the intestinal digestion and absorption of lipids and fat-soluble vitamins, but also as signalling molecules involved in regulating many physiological functions through interaction with their specific nuclear and membrane receptors.^1^ BAs, synthesised from cholesterol in the liver, are conjugated to form primary BAs or metabolised by gut microbes to form secondary BAs. Two primary BAs in humans, cholic acid (CA) and chenodeoxycholic acid (CDCA), are produced in approximately equal amounts. The conjugation of the side-chain terminal carboxylic acid with the amino acids glycine or taurine generates the four primary human BAs, i.e., glycocholic acid (GCA), taurocholic acid (TCA), glycochenodeoxycholic acid (GCDCA), and taurochenodeoxycholic acid (TCDCA). The bile salt export pump (BSEP), an ATP-binding cassette (ABC) protein (encoded by *ABCB11*) expressed at the canalicular membrane of hepatocytes, is the major transporter responsible for BA biliary secretion.^2^ Once their role in digestion is fulfilled, most BA molecules are efficiently recovered from the intestine; only 5% of the BA pool is lost daily in the faeces.^3^ Anaerobes generate the secondary BAs deoxycholic acid (DCA) and lithocholic acid (LCA) from CA and CDCA, respectively, by 7α-dehydroxylation through a multistep enzymatic pathway.

Besides the predominant role of the Na^+^/taurocholate cotransporting polypeptide (NTCP) in BA uptake by the liver, organic anion transporting polypeptides (OATPs) are also involved in BA recovery from the hepatic sinusoidal blood. These transporters play a more critical role in the uptake of other endogenous compounds, such as steroid sulfates and numerous drugs.^4^ Subsequently, the biotransformation of a wide spectrum of drugs by phase I and phase II metabolism occurs within hepatocytes. Several members of the ABC superfamily of transporters also mediate the efflux of xenobiotics and their metabolites into bile or back to the sinusoidal blood for subsequent renal excretion.^5^ The liver thus has a central role in the biotransformation and clearance of a large number of drugs.

Drug-induced liver injury (DILI) is an unexpected adverse effect occurring occasionally in response to recommended-dose drug or dietary supplement intake. Outcomes can be severe (liver failure, death), necessitating the development of suitable biomarkers for identification and stratification of cases. A recent study demonstrated that shared genetic risk factors (polygenic risk score) underpinned DILI due to new (fasiglifam), and currently-used (amoxicillin-clavulanate and flucloxacillin) medications as well as hepatotoxicity in primary hepatocytes and stem cell-derived organoids from multiple donors treated with over ten different drugs.^6^ Pathway analysis highlighted intrahepatic BAs to be an important initiator of processes previously implicated in DILI.^6^ Earlier studies reported elevation of total serum BAs and prognostic utility of serum GCA (for liver injury related to solvents and acetaminophen (APAP), respectively).^7^^,^^8^ Additional studies have found elevation of specific serum BA species in patients with APAP overdose,^9^ DILI due mostly to herbals/unknown agents in traditional medicines^10^^,^^11^ or medications and supplements,^12^ and have described associations with liver injury severity^10^^,^^11^ or outcomes.^8^^,^^12^ However, a recent systematic review concluded BAs were only suitable biomarkers for intrahepatic cholestasis of pregnancy.^13^ Further, a systematic review highlighted the current lack of non-genetic biomarkers for DILI clinical outcomes.^14^ Since DILI is also the most common adverse reaction that leads to the termination of clinical trials during drug development,^15^ the search for new and reliable prognostic biomarkers is crucial.^16^ This current study aims to explore whether changes in serum BA concentrations and profiles can serve as useful biomarkers for diagnostic and prognostic purposes in DILI. Although BA profiles were unable to distinguish DILI from acute nonDILI group, we have focussed on evaluating BA profile as a prognostic biomarker to identify those patients with acute DILI who progress, following first presentation, and develop acute liver failure requiring transplantation or death, to inform clinical decision-making on hospital admission and listing for transplantation.

## Methods

### Ethics

Ethical approvals were obtained from local Ethical Review Authorities (Supplemental Table S1). Studies were conducted according to the Declaration of Helsinki (Hong Kong Amendment) and Good Clinical Practice (European guidelines) with all participants providing written informed consent or with written informed consent from a personal consultee in specified circumstances when participants lack capacity to give informed consent.

### Study design and population

A nested case–control observational study design was devised to prospectively identify and enrol a cohort of patients with acute liver injury at presentation at secondary care centres in six European countries through attendance for standard clinical care pathways.^17^ Patients aged 18 or over where there is suspicion of DILI and meeting the criteria for DILI, as defined by Aithal et al.^18^ and endorsed by the EASL DILI guidelines,^19^ were prospectively recruited. Patients were assessed clinically and through investigations (laboratory tests and imaging) and sub-grouped as DILI (‘cases’) or acute nonDILI (‘controls’), due to alternate causes. Both cases and controls were followed up to recovery or death or transplantation, when possible. Sex and ethnicity were self-reported. Outcomes at 6 months were recorded. Blood samples were obtained from both groups at time of acute liver injury when DILI criteria (alanine aminotransferase (ALT) ≥5x upper limit of normal (ULN) or alkaline phosphatase (ALP) ≥2x ULN, or ALT ≥3x ULN + serum total bilirubin (TBIL) >2x ULN) are met. In addition, samples from healthy volunteers (HV) were collected. The inclusion and exclusion criteria for each study cohort are detailed in Suppemental Table S2. Initially-suspected patients with acute DILI were classified after adjudication by an expert panel, as confirmed DILI or with a nonDILI aetiology explaining the clinical manifestation,^19^ DILI was phenotyped and severity graded as recommended by the DILI phenotype standardisation project.^18^ The clinical definitions applied were: i) ‘Severe’, when the case met the defined DILI acute injury criteria and TBIL was ≥2x ULN; there were symptoms of either encephalopathy, ascites or elevation of International Normalised Ratio (INR) ≥1.5 (in patients where INR was not reported, a value calculated as prothrombin time (PT)/(PT ULN) was used); or other clinical assignment of acute liver failure; ii) ‘Moderate’, where TBIL ≥2x ULN but there were no symptoms associated with severe injury/acute liver failure; and iii) ‘Mild’, when patients meet the defined DILI acute injury criteria but TBIL<2x ULN. Increases in the severity scale after the initial sampling visit were termed ‘progression’. The pattern of injury was classified based on R value ((ALT/ULN)/(ALP/ULN)) at first testing, as cholestatic (R ≤ 2), hepatocellular (R ≥ 5) and mixed (R > 2 and < 5).^18^ Patients who received ursodeoxycholic acid medications were excluded (6 cases).

### Biomarker analysis

Plasma concentrations of 22 molecular BA species were determined by liquid chromatography tandem mass spectrometry (6420 Triple Quad LC/MS, Agilent Technologies, Santa Clara, CA) using an adaptation^20^ of a previously described method^21^ (see Supplemental Methods). For patients, standard clinical biomarkers were determined at time of research visit as part of clinical care. For HV, these biomarkers were quantified in serum samples using standard clinical biochemistry assays (Cobas 6000 system, Roche) at MLM Medical Labs (Mönchengladbach, Germany). All analysis was done by researchers blinded to the study group.

### Statistics

Analysis of variance (ANOVA) was performed with demographic factors age, sex and BMI as covariates. The BA and model for end-stage liver disease (MELD) values were transformed on the logarithmic scale. Post-hoc Tukey's HSD method was used for multiple comparison adjustment. Fisher's exact test was employed to determine whether there were statistically significant associations between sex and cohort of patients (HV/DILI/NonDILI), different severity levels, being progression/severe outcome patients. The significance level (α) was set to 5%. Prognostic performance was determined by receiver operator characteristic (ROC) curve analyses (see Supplemental Methods). Area under the curve (AUC) of 0.75 or higher was considered an acceptable discrimination. The optimal threshold of each biomarker was determined using Youden's method and back transformed from the logarithmic scale to its initial unit. Bivariate regression and integrated discrimination improvements (IDI), and Decision Curves were also analysed considering the synergy of two biomarkers.^22^ Additional details are provided in the Supplemental Methods. Since DILI is rare and there are no established biomarkers, the cohort size of this study was informed by previous studies,^7^^,^^8^^,^^12^ and followed the international guidelines of the Clinical and Laboratory Standards Institute (CLSI) CLSI 28-A3c recommendations.

### Role of funders

In the process of developing the TransBioline Project Proposal, the funders, IMI2, were consulted. The funders did not specify the study design for this investigation and had no role in recruitment, data collection, data analyses, interpretation, writing of the manuscript or decision to submit for publication.

## Results

**Fig. 1:**
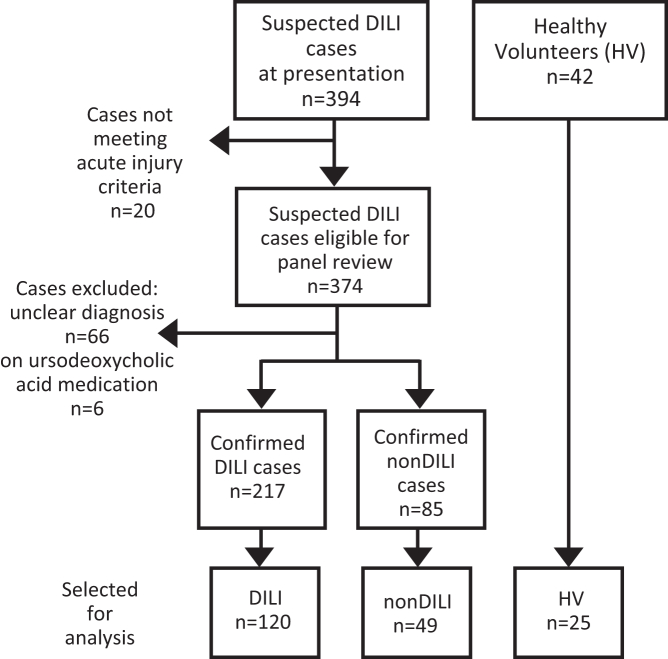
**Flow diagram illustrating derivation of nested cohorts.** The inclusion criteria are specified in Supplemental Table S1 and selection of cases for analysis in batches is described in Supplemental Methods (all cases with outcomes/progression were selected). DILI, drug-induced liver injury; HV, healthy volunteers.

**Fig. 2:**
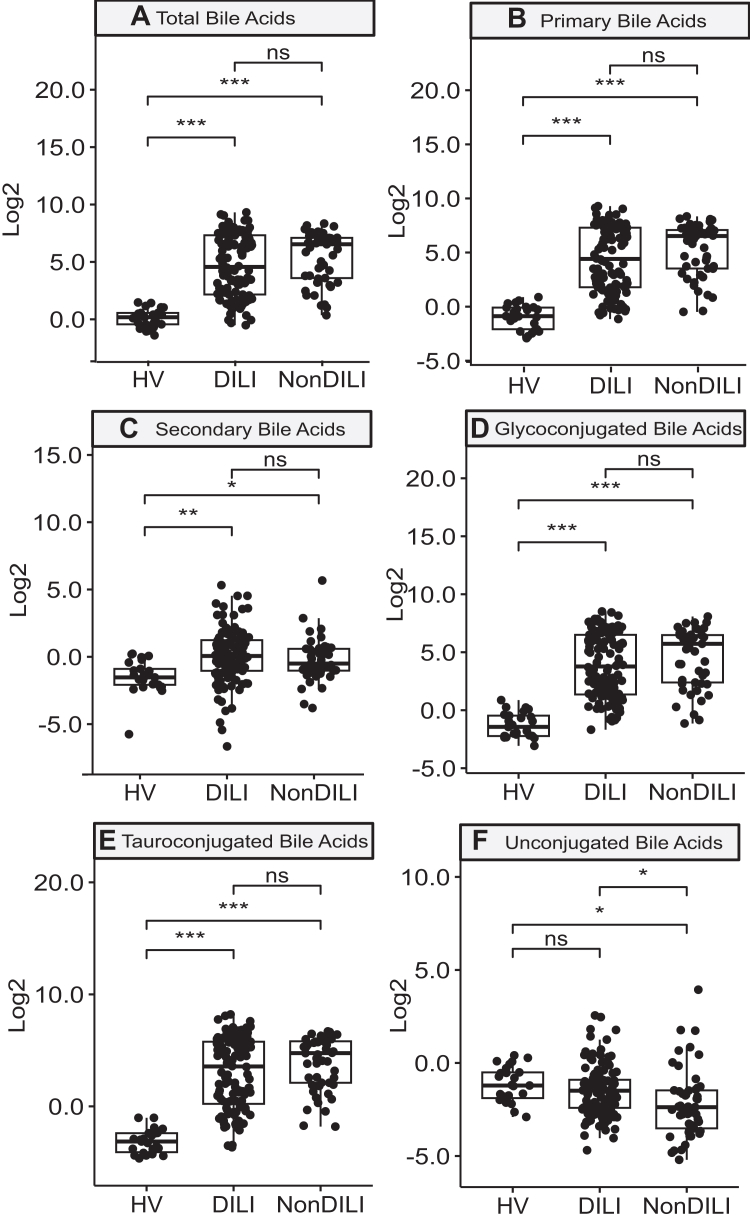
**Levels of selected plasma bile acids in healthy volunteers (HV, n = 25) and patients with acute drug-induced liver injury (DILI, n = 120) or alternate causes of acute liver injury (nonDILI, n = 49).** Log value of μM concentration is displayed. p values (ANOVA) adjusted by Tukey method: ∗∗∗≤0.001; ∗∗≤0.005, ∗≤0.05; ns, not significant.

The derivation of the participant groups analysed is illustrated in Fig. 1. Demographic information is shown in Table 1 (Supplemental Table S3 shows inter-centre analysis); there are differences in age, BMI and aspartate aminotransferase (AST) between the patient cohorts reflecting the variations in patients recruited in this study design. Levels of BA types for the participant groups and sub-groups are shown in Tables 2 and 3. Individual BA levels are reported in Supplemental Tables S4 and S5. Total plasma BA levels in patients with DILI were significantly elevated, with mean values approaching 100 μM, which were approximately 75 times higher than those observed in the HV group (Fig. 2, Table 2). This occurrence was not exclusive to DILI, as patients in the nonDILI group also displayed marked hypercholanemia. The increased total BA concentrations were primarily due to a rise in primary BAs (Fig. 2B). Although secondary BAs were also significantly higher in patients with DILI, they only reached mean values of approximately 5 times those found in HV (Fig. 2C; Table 2). Regarding BA conjugation, both glyco- (Fig. 2D) and tauroconjugated forms (Fig. 2E) were elevated in the plasma of patients with liver damage, affecting both DILI and nonDILI groups. In contrast, patients with DILI did not display an increase in unconjugated BAs (Fig. 2F).

**Table 1:** Characteristics of study groups.

Mean ± SD (Min–Max)	Healthy volunteers vs. nonDILI and DILI			DILI: severity groups			DILI: progression groups		DILI: death/transplant outcome	
	HV n = 25	NonDILI n = 49	DILI n = 120	Mild n = 56	Moderate n = 46	Severe n = 18	No progression n = 90	Progressed n = 28	No n = 101	Yes n = 19
Sex Male (n)	40% (10)	55% (27)	42% (50)	38% (21)	52% (24)	28% (5)	41% (37)	39% (11)	42% (42)	42% (8)
Ethnicity										
Caucasian	100% (25)	98% (48)	90% (107)	89% (49)	96% (44)	78% (14)	93% (83)	82% (23)	91% (91)	84% (16)
Other	0% (0)	2.0% (1)	10% (12)	11% (6)	4% (2)	22% (4)	7% (6)	18% (5)	9% (9)	16% (3)
Age (years)	**48 ± 13 (19–66)**	**55 ± 19**^a^**(20–87)**	**59 ± 17**^a^**^,^**^b^**(21–89)**	**58 ± 16 (23–84)**	**63 ± 17**^c^**(24–89)**	**49 ± 15**^c^**^,^**^d^**(21–76)**	59 ± 17 (21–89)	59 ± 18 (24–85)	**58 ± 17 (21–89)**	**60 ± 17**^e^**(24–85)**
BMI	**24.8 ± 3.1 (19.5–30.3)**	**28.7 ± 7.7**^a^**(18.2–55.4)**	**27.0 ± 5.5**^a^**^,^**^b^**(15.1–42.3)**	**26.3 ± 5.6 (15.1–39.8)**	**27.5 ± 4.6**^c^**(20.0–37.7)**	**28.5 ± 7.0**^c^**^,^**^d^**(21.6–42.3)**	27.1 ± 5.5 (18.5–42.3)	26.8 ± 5.7 (15.1–41.5)	27.1 ± 5.4 (18.5–42.3)	26.9 ± 5.9 (15.1–41.5)
TBIL (mg/dL)	**1 ± 0 (0–1)**	**11 ± 9**^a^**(1–36)**	**9 ± 1**^a^**(1–44)**	**2 ± 5 (1–38)**	**12 ± 10**^c^**(2–44)**	**20 ± 8**^c^**^,^**^d^**(5–31)**	**7 ± 8 (1–38)**	**14 ± 14**^e^**(1–44)**	**6 ± 8 (1–38)**	**20 ± 13**^e^**(1–44)**
Creatinine (mg/dL)	**0.75 ± 0.11 (0.54–0.96)**	**1.13 ± 0.49**^a^**(1.00–4.00)**	**1.13 ± 0.37**^a^**(1.00–4.00)**	**1.07 ± 0.20 (1.00–2.00)**	**1.16 ± 0.51 (1.00–4.00)**	**1.27 ± 0.37**^c^**^,^**^d^**(1.00–2.00)**	**1.09 ± 0.35 (1.00–4.00)**	**1.25 ± 0.43**^e^**(1.00–2.54)**	**1.09 ± 0.34 (1.00–4.00)**	**1.32 ± 0.47**^e^**(1.00–2.54)**
ALT (U/L)	**20 ± 10 (9–44)**	**893 ± 826**^a^**(59–4339)**	**629 ± 56**^a^**(70–3207)**	512 ± 430 (70–2320)	632 ± 482 (81–2224)	983 ± 932 (117–3207)	**553 ± 477 (70–3207)**	**862 ± 747**^e^**(95–2799)**	592 ± 517 (70–3207)	832 ± 787 (95–2799)
AST (U/L)	**22 ± 5 (14–32)**	**753 ± 952**^a^**(51–6073)**	**409 ± 496**^a^**^,^**^b^**(35–3792)**	**258 ± 243 (35–1207)**	**349 ± 242**^c^**(74–926)**	**1104 ± 948**^c^**^,^**^d^**(177–3792)**	**308 ± 309 (38–1972)**	**784 ± 811**^e^**(35–3792)**	**334 ± 320 (35–1972)**	**915 ± 995**^e^**(101–3792)**
ALP (U/L)	NA	360 ± 386 (86–2107)	368 ± 362 (48–1940)	**303 ± 265 (48–1426)**	**523 ± 467**^c^**(126–1940)**	186 ± 79 (81–397)	381 ± 361 (48–1724)	333 ± 382 (95–1940)	369 ± 350 (48–1724)	367 ± 437 (98–1940)
Albumin (g/dL)	**4 ± 0.2 (4–5)**	**3 ± 1**^a^**(2–4)**	**3 ± 1**^a^**(2–5)**	**3.61 ± 0.51 (2.60–4.60)**	**3.27 ± 0.77 (1.90–4.60)**	**2.60 ± 0.3**^c^**^,^**^d^**(2.00–3.10)**	**3.49 ± 0.63 (2.08–4.60)**	**2.76 ± 0.62**^e^**(1.90–4.50)**	**3.50 ± 0.63 (2.08–4.60)**	**2.59 ± 0.40**^e^**(1.90–3.50)**
MELD score	NA	16 ± 7 (6–47)	14 ± 8 (6–43)	**8 ± 3 (6–22)**	**16 ± 5**^c^**(9–30)**	**28 ± 7**^c^**^,^**^d^**(21–43)**	**12 ± 6 (6–26)**	**21 ± 11**^e^**(7–43)**	**12 ± 5 (6–26)**	**26 ± 10**^e^**(7–43)**

Missing data: ethnicity was unknown in 1 DILI case; progression was unknown in 2 DILI cases. For Sex and Ethnicity variables, Fisher's exact test was performed; For Age and BMI, one way Anova was performed. For biomarkers, one way ANOVA was performed and adjusted by demographic factors (age, BMI, and sex).

ALT, alanine aminotransferase; AST, aspartate aminotransferase; ALP, alkaline phosphatase; BMI, body mass index; DILI, drug-induced liver injury; HV, healthy volunteers; MELD, Model for End-Stage Liver Disease; NA, not applicable; SD, standard deviation; TBIL, total bilirubin.

Tukey adjusted p value for three groups comparison or T-test p value for two group comparison (bold values indicates statistical significance <0.05):

a nonDILI or DILI group is significantly different from HV group.

b DILI group is significantly different from nonDILI group.

c Moderate or Severe group is significantly different from Mild group.

d Severe group is significantly different from Moderate group.

e Progressor group or Death/transplantation group is significantly different from Non-progressor or without severe outcome, respectively.

**Table 2:** Levels of bile acid types in study cohorts.

Bile acids μM mean ± SD (Min–Max)	Cohort			DILI severity groups		
	HV n = 25	NonDILI n = 49	DILI n = 120	Mild n = 56	Moderate n = 46	Severe n = 18
Total BAs	**1.24 ± 0.67 (0.38–2.77)**	**94.17 ± 87.97**^a^**(1.28–324.91)**	**90.59 ± 122.66**^a^**(0.70–638.23)**	**22.49 ± 52.40 (0.70–256.09)**	**136.56 ± 137.98**^c^**(3.98–638.23)**	**184.97 ± 124.94**^c^**(0.96–564.54)**
Primary BAs	**0.67 ± 0.46 (0.13–1.84)**	**91.36 ± 86.49**^a^**(0.72–323.91)**	**87.34 ± 119.95**^a^**(0.45–624.80)**	**19.61 ± 47.34 (0.45–214.04)**	**132.85 ± 134.80**^c^**(2.78–624.80)**	**181.74 ± 123.33**^c^**(0.90–559.61)**
Secondary BAs	**0.47 ± 0.33 (0.02–1.17)**	**2.30 ± 7.24**^a^**(0.07–50.85)**	**2.60 ± 5.13**^a^**(0.01–40.02)**	2.20 ± 5.57 (0.01–40.02)	3.10 ± 4.93 (0.02–23.14)	2.54 ± 4.29 (0.03–15.57)
Unconjugated BAs	0.55 ± 0.35 (0.13–1.33)	**0.74 ± 2.25 (0.03–15.34)**	**0.61 ± 0.89**^b^**(0.04–5.88)**	0.61 ± 0.81 (0.07–5.53)	0.65 ± 1.10 (0.06–5.88)	0.49 ± 0.42 (0.04–1.41)
Conjugated BAs	**0.69 ± 0.51 (0.24–2.33)**	**93.43 ± 88.17**^a^**(0.86–324.00)**	**89.97 ± 122.70**^a^**(0.40–638.00)**	**21.88 ± 52.48 (0.40–255.62)**	**135.90 ± 138.12**^c^**(2.93–638.00)**	**184.46 ± 124.70**^c^**(0.92–563.08)**
Glycoconjugated BAs	**0.54 ± 0.43 (0.12–1.83)**	**60.60 ± 61.58**^a^**(0.45–270.65)**	**54.14 ± 74.33**^a^**(0.31–366.81)**	**14.11 ± 35.79 (0.31–196.89)**	**74.49 ± 78.91**^c^**(1.49–366.81)**	**126.67 ± 77.94**^c^**(0.58–346.34)**
Tauroconjugated BAs	**0.15 ± 0.12 (0.04–0.49)**	**32.83 ± 30.21**^a^**(0.29–103.49)**	**35.83 ± 53.78**^a^**(0.08–296.15)**	**7.76 ± 18.86 (0.08–95.50)**	**61.41 ± 67.04**^c^**(0.83–296.15)**	**57.79 ± 48.16**^c^**(0.34–216.74)**
Ratio (CA + DCA)/(CDCA + LCA)	1.67 ± 0.98 (0.19–3.88)	1.77 ± 0.97 (0.32–4.41)	1.81 ± 2.18 (0.32–21.66)	**1.24 ± 0.62 (0.41–3.33)**	**2.84 ± 3.19**^c^**(0.32–21.66)**	**0.94 ± 0.50**^c^**(0.41–2.05)**
Ratio CA/DCA	**2.12 ± 5.43 (0.10–27.45)**	**230.35 ± 526.52**^a^**(0.27–2767.73)**	**221.43 ± 458.78**^a^**(0.14–2956.34)**	**67.83 ± 297.81 (0.14–1614.82)**	**392.04 ± 591.62**^c^**(1.93–2956.34)**	**263.30 ± 318.70**^c^**(4.36–898.20)**
Ratio CDCA/LCA	**11.98 ± 36.58 (0.58–186.29)**	**163.59 ± 206.83**^a^**(1.22–1097.91)**	**108.05 ± 182.89**^a^**(0.58–884.39)**	**35.03 ± 61.23 (0.72–323.97)**	**87.06 ± 156.82**^c^**(0.58–776.82)**	**388.88 ± 233.75**^c^^**,**^^d^**(15.76–884.39)**
Ratio glyco/tauroconjugated BAs	**5.19 ± 5.60 (0.94–26.64)**	**1.89 ± 0.95**^a^**(0.29–5.07)**	**2.50 ± 1.86**^a^**(0.27–11.60)**	**3.27 ± 2.25 (0.27–11.60)**	**1.61 ± 1.14**^c^**(0.33–4.67)**	2.40 ± 0.62 (1.60–3.80)
Ratio primary/secondary BAs	**5.49 ± 19.52 (0.37–99.00)**	**124.42 ± 160.48**^a^**(0.49–727.09)**	**83.34 ± 138.06**^a^**(0.50–813.03)**	**18.99 ± 53.07 (0.50–306.12)**	**97.79 ± 103.10**^c^**(2.36–421.61)**	**246.65 ± 231.81**^c^^**,**^^d^**(15.70–813.03)**
Ratio 12αOH/non-12αOH	1.52 ± 0.92 (0.17–3.76)	1.73 ± 0.93 (0.32–4.31)	1.76 ± 2.18 (0.32–21.58)	**1.16 ± 0.60 (0.36–3.17)**	**2.82 ± 3.18**^c^**(0.32–21.58)**	**0.93 ± 0.49**^d^**(0.41–2.04)**

BA, bile acids; CA, cholic acid; CDCA, chenodeoxycholic acid; DCA, deoxycholic acid; DILI, drug-induced liver injury; HV, healthy volunteer; LCA, lithocholic acid; 12αOH, 12alpha-hydroxylated BAs.

Tukey adjusted p value (ANOVA) (bold values indicates significance <0.05):

a nonDILI or DILI group is significantly different from HV group.

b DILI group is significantly different from nonDILI group (value shown in bold).

c Moderate or Severe group is significantly different from Mild group.

d Severe group is significantly different from Moderate group.

**Table 3:** Levels of bile acid types in study groups.

Bile acids μM mean ± SD (Min–Max)	DILI: progression group		DILI: outcome death/liver transplantation	
	No progression n = 90	Progressed n = 28	No n = 101	Yes n = 19
Total BAs	**85.72 ± 125.11 (0.80–638.23)**	**112.39 ± 116.57**^a^**(0.96–564.54)**	**81.57 ± 120.74 (0.70–638.23)**	**138.52 ± 124.88**^a^**(0.96–564.54)**
Primary BAs	**82.35 ± 121.91 (0.45–624.80)**	**109.31 ± 115.44**^a^**(0.90–559.61)**	**78.21 ± 117.54 (0.45–624.80)**	**135.90 ± 124.13**^a^**(0.90–559.61)**
Secondary BAs	2.65 ± 5.62 (0.02–40.02)	2.57 ± 3.41 (0.03–13.32)	2.69 ± 5.46 (0.01–40.02)	2.08 ± 2.84 (0.03–12.19)
Unconjugated BAs	0.64 ± 0.98 (0.08–5.88)	0.52 ± 0.52 (0.04–2.39)	0.64 ± 0.95 (0.07–5.88)	0.44 ± 0.39 (0.04–1.41)
Conjugated BAs	**85.07 ± 125.20 (0.40–638.00)**	**111.85 ± 116.48**^a^**(0.92–563.08)**	**80.93 ± 120.83 (0.40–638.00)**	**138.05 ± 124.63**^a^**(0.92–563.08)**
Glycoconjugated BAs	**49.71 ± 74.03 (0.31–366.81)**	**72.04 ± 75.44**^a^**(0.58–346.34)**	**47.49 ± 71.69 (0.31–366.81)**	**89.54 ± 80.00**^a^**(0.58–346.34)**
Tauroconjugated BAs	35.36 ± 56.63 (0.09–296.15)	39.81 ± 45.48 (0.34–216.74)	**33.44 ± 54.49 (0.08–296.15)**	**48.52 ± 49.21**^a^**(0.34–216.74)**
Ratio (CA + DCA)/(CDCA + LCA)	**2.00 ± 2.41 (0.32–21.66)**	**1.26 ± 1.11**^a^**(0.41–5.02)**	**1.92 ± 2.30 (0.32–21.66)**	**1.19 ± 1.21**^a^**(0.41–5.02)**
Ratio CA/DCA	223.24 ± 491.89 (0.14–2956.34)	229.88 ± 356.48 (0.63–1160.51)	205.46 ± 469.65 (0.14–2956.34)	306.32 ± 396.34 (0.63–1160.51)
Ratio CDCA/LCA	81.63 ± 151.22 (0.58–776.82)	188.22 ± 247.11 (1.69–884.39)	**78.11 ± 146.08 (0.58–776.82)**	**267.21 ± 266.09**^a^**(1.69–884.39)**
Ratio glyco/tauroconjugated BAs	2.52 ± 1.98 (0.27–11.60)	2.29 ± 1.28 (0.43–6.68)	2.57 ± 1.99 (0.27–11.60)	2.14 ± 0.84 (0.52–3.61)
Ratio primary/secondary BAs	67.47 ± 107.03 (0.50–555.70)	137.26 ± 205.50 (1.42–813.03)	**62.23 ± 102.29 (0.50–555.70)**	**195.56 ± 228.24**^a^**(1.42–813.03)**
Ratio 12αOH/non12αOH BAs	**1.94 ± 2.41 (0.32–21.58)**	**1.24 ± 1.11**^a^**(0.41–4.99)**	**1.87 ± 2.30 (0.32–21.58)**	**1.17 ± 1.21**^a^**(0.41–4.99)**

BA, bile acids; CA, cholic acid; CDCA, chenodeoxycholic acid; DCA, deoxycholic acid; DILI, drug-induced liver injury; LCA, lithocholic acid; SD, standard deviation; 12**α**OH, 12alpha-hydroxylated BAs.

ANOVA T-test p value (bold values indicates significance <0.05):

a Progressor group or Death/transplantation group is significantly different from Non-progressor or without death/transplantation outcome, respectively.

**Fig. 3:**
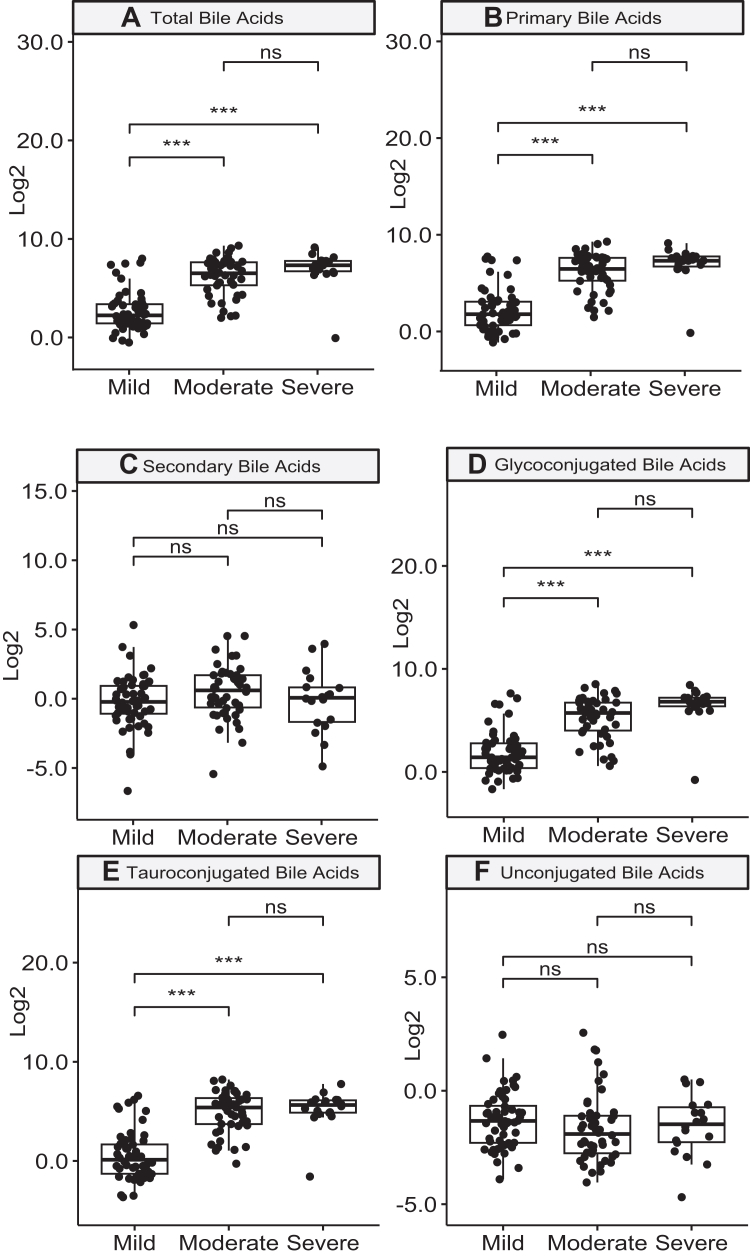
**Levels of selected bile acids in patients with acute drug-induced liver injury (DILI) stratified according to liver injury severity.** Mild (n = 56), Moderate (n = 46), Severe (n = 18) DILI cases. Log value of μM concentration is displayed. p values (ANOVA) adjusted by Tukey method, ∗∗∗≤0.001; ∗∗≤0.005; ∗≤0.05; ns, not significant.

The degree of hypercholanemia was associated with the severity of liver injury. Thus, when patients with DILI were classified according to the presence of mild, moderate, or severe liver damage, plasma concentrations of total BAs, as well as primary BAs and both glycine- and taurine-conjugated species, were significantly higher in moderate and severe DILI compared to mild DILI (Fig. 3). Although there was a trend showing a further increase in these parameters when comparing severe to moderate DILI, this increase did not reach statistical significance. No severity-dependent differences were noted for secondary (Fig. 3C) and unconjugated BA species (Fig. 3F). Similar results were observed when examining severity in patients with acute nonDILI (Supplemental Fig. S1). Further, comparison of BAs between cholestatic and hepatocellular injury type cases showed no significant differences in levels of total BAs, primary BAs, unconjugated BAs, tauroconjugated BAs and glycoconjugated BAs. Secondary BAs were significantly decreased in DILI cholestatic cases, consistent with impaired function of BSEP (Supplemental Figs. S2 and S3).

**Fig. 4:**
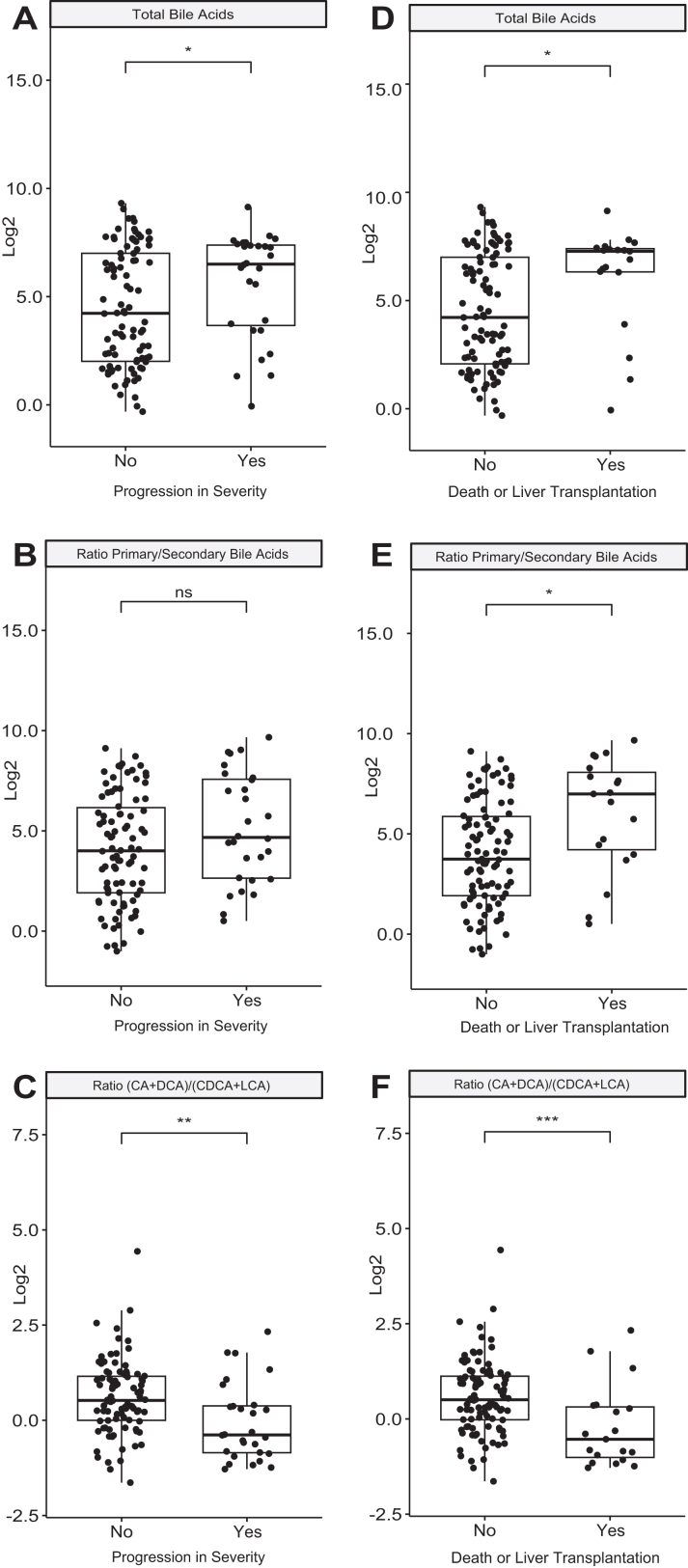
**Levels of selected bile acids in patients with acute drug-induced liver injury (DILI). A–C Stratified according to progression in severity (yes, n = 28) or no progression (no, n = 90). D–F stratified according to outcome of death/transplantation (yes, n = 19) or not (no, n = 101).** Log value of μM concentration or log of ratio is displayed. T-test p values (ANOVA) ∗∗∗≤0.001,∗∗≤0.005, ∗≤ 0.05, ns, not significant. CA, cholic acid; DCA deoxycholic acid; CDCA, chenodeoxycholic acid; LCA, lithocholic acid.

To investigate the potential prognostic value of circulating BA levels and profile in patients with DILI, patients were classified into progressors (n = 28) and non-progressors (n = 90), based on whether their condition worsened on the severity scale^18^ following the serum sampling. Progressors exhibited higher levels of total BAs (Fig. 4, Table 3). This was also notable when comparing patients who experienced worse outcomes (death or need for liver transplantation, n = 19) with those who did not experience such progression (n = 101) (Fig. 4, Table 3). Regarding individual BA molecular species, plasma concentrations of GCA, GCDCA, TCDCA, and CDCA were significantly higher in patients who progressed to death or transplantation (Supplemental Table S5). When calculating the ratio of primary to secondary BAs, this was also increased in progressors and in those with death/transplantation outcomes (Fig. 4, Table 3). Finally, we found that the ratio between CA plus its secondary derivative, deoxycholic acid (DCA), to CDCA plus its secondary derivative lithocholic acid (LCA), which reflects the balance between the two major BA families synthesised by hepatocytes, was significantly reduced in DILI progressors (Fig. 4, Table 3) and in those with death/transplantation outcome (Fig. 4, Table 3, Supplemental Table S6).

**Fig. 5:**
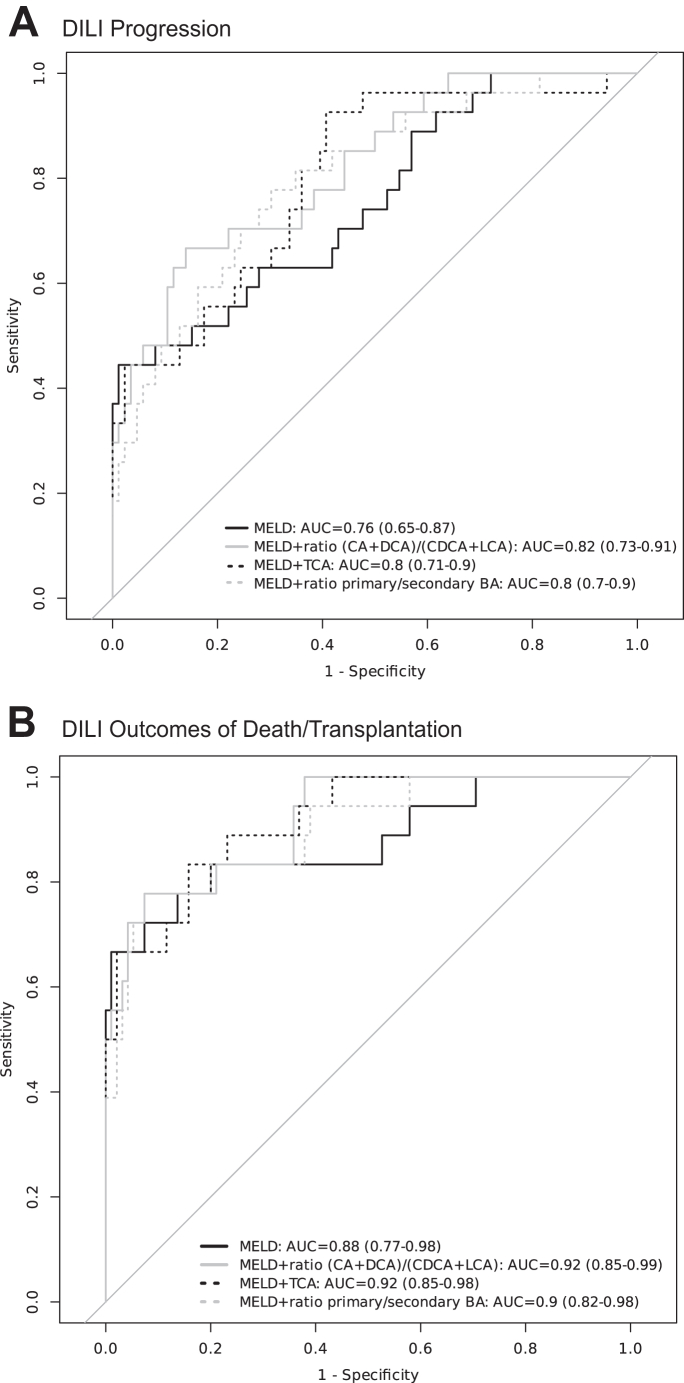
**Receiver operator characteristic (ROC) curves for best performing bile acid biomarkers combined with Model of End-stage Liver Disease (MELD) score. A** For DILI cases with progression in severity (n = 118). **B** DILI cases with outcome of death/transplantation (n = 120). The AUC 95% CI are shown in parentheses. AUC, Area under ROC curve; BA, bile acids; CA, cholic acid; CI, confidence interval; DCA deoxycholic acid; CDCA, chenodeoxycholic acid; LCA, lithocholic acid; TCA, taurocholic acid.

The performance of BAs as a prognostic biomarker for death/transplantation was compared to the MELD score (Supplemental Table S7). Although MELD had a sensitivity of 0.99 the specificity was only 0.67. Tauroursodeoxycholic (TUDCA) and sulfolithocholic acid (SLCA) had the best specificity (0.84). MELD also had the highest AUC for DILI progression (Supplemental Table S8). These findings prompted us to investigate whether BA species and/or ratios could improve the prognostic value of the MELD score in DILI. The results of bivariate analysis are shown in Supplemental Tables S7 and S8 and in Fig. 5.

The best results were obtained when combining the MELD score with the (CA + DCA) to (CDCA + LCA) ratio, considering both all DILI progressors and patients with the worst outcomes (Fig. 5). The combination of the MELD score with the ratio of primary to secondary BAs, as well as with particular BA species like TCA, also enhanced the AUC compared to the MELD score alone suggesting incremental value on IDI analysis (Supplemental Tables S9 and S10). Further, Decision Curve Analysis supports the potential for improvement in clinical application in identifying patients for immediate hospital admission (‘decision to admit’; Supplemental Figs. S4 and S5).

## Discussion

**Fig. 6:**
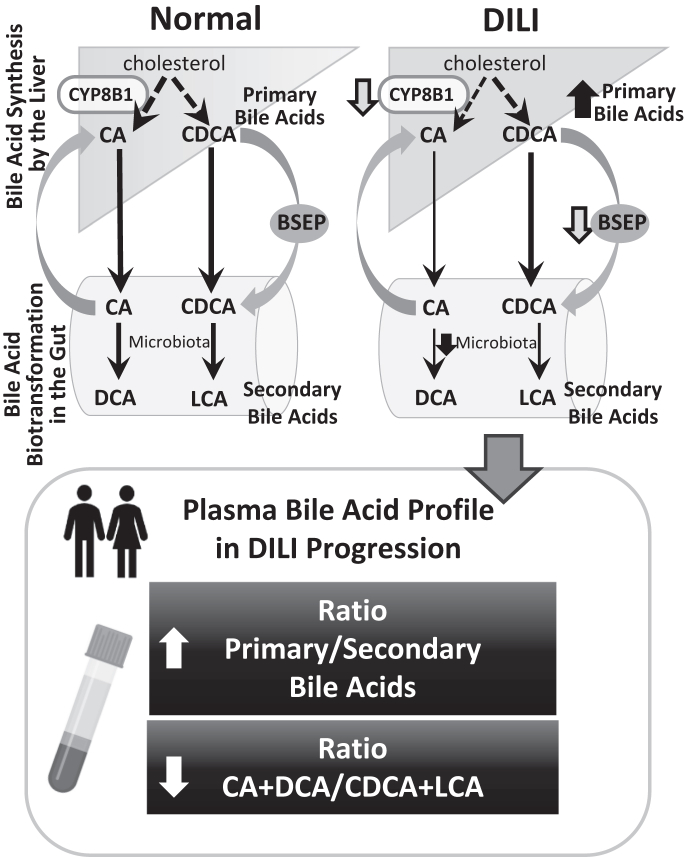
**Putative mechanisms that determine the changes in bile acid profiles in association with DILI and its biochemical manifestations.** CYP8B1 activity is decreased in presence of inflammatory cytokines and is decreased during liver injury,^24^^,^^25^ restricting generation of CA. Dysbiosis arises during DILI limiting production of secondary BA.^23^^,^^26^ BSEP inhibition or deficiency occurring during some DILI episodes,^27^, ^28^, ^29^ also contributes to impaired BA homoeostasis. CA, cholic acid; CDCA, chenodeoxycholic acid; DCA deoxycholic acid; DILI, drug-induced liver injury; LCA, lithocholic acid.

This prospective, multicenter, longitudinal cohort study with a nested case–control design has demonstrated that in patients presenting with clinical or biochemical manifestations of acute liver injury (due to DILI or nonDILI), higher values of total, primary, and conjugated BAs at presentation, are associated with liver injury that is likely to progress in severity. Although DILI primarily affects the liver, recent reports indicate that patients with DILI experience a disrupted gut–liver axis characterised by dysbiosis, increased intestinal permeability, and impaired BA homoeostasis.^23^ This may be mediated by decreased conversion of deconjugated primary BAs to deconjugated secondary BAs as a consequence of altered gut microbiota composition (Fig. 6).^26^

Additionally, patients with DILI who died or required liver transplantation had lower ratio of CA + DCA to CDCA + LCA compared to the rest of the DILI group, indicating a relatively higher proportion of CDCA and its secondary LCA compared to CA and its secondary DCA. The higher proportion of combined CDCA + LCA is more hydrophobic, thus, a stronger detergent and more toxic than CA + DCA, which could explain the association with poor outcomes in DILI. The ratio of CA + DCA to CDCA + LCA may decrease due to reduced activity of the enzyme CYP8B1, which is responsible for the 12α hydroxylation of the steroid ring necessary for CA synthesis, but is not necessary for CDCA synthesis.^30^ CYP8B1 activity, which is exclusive to the liver, is compromised in liver injury.^24^ Moreover, inflammatory cytokines inhibit *CYP8B1* transcription,^25^ which might decrease enzyme activity, leading to apparent preferential CDCA synthesis in DILI (Fig. 6).

Circulating BA concentrations have been used effectively in assessing severity and predicting outcomes in patients with intrahepatic cholestasis of pregnancy^31^ as well as used as a biomarker assessing response to treatment with ileal bile acid transporter inhibitors in progressive familial intrahepatic cholestasis.^32^ Elevation of serum BAs (mainly TCA, TCDCA, GCA and GCDCA) has been reported in a large cohort of patients with various liver impairments and distinct profiles associated with different conditions; only slight differences in BA signatures were found in patients with APAP overdose compared to other liver disorders.^9^ Luo et al. used clinical chemistry based criteria to define acute liver injury and then reported the performance of BAs to identify liver injury involving 10 different etiologies; by design, clinical application of BAs could not be evaluated in this study. Mireault et al. also described changes in BA profiles associated with APAP related acute liver failure.^33^ Analysis of 1589 serum metabolites in patients with DILI and healthy volunteers and partial least squares discriminant analysis suggested that CA and DCA had potential as biomarkers for progression to chronic disease.^12^ Moreover, in a cross-sectional cohort study, the elevation of serum concentrations of GCDCA, NorCA and TCDCA was shown to correlate with the severity of idiosyncratic DILI.^11^ This study included patients in which 64%–87% had traditional Chinese medicine or herbal and dietary supplements as the underlying aetiology. Although their study described BA profile according to the severity, there were none who died or had transplantation, which suggests that full spectrum of severity was not represented. Increased serum levels of CDCA and DCA have also been recently reported in patients with DILI (15 mild and 23 severe cases, none with death or transplantation) when compared to levels in healthy volunteers.^10^

In the present study, we also found elevations in circulating BA levels to be associated with the severity of DILI at presentation. Primary conjugated BAs were responsible for this increase, while secondary unconjugated BAs, produced by the intestinal microbiota's biotransformation of the former, were only moderately increased or even unchanged in patients with DILI. Consistent with our findings, a study analysing the BA serum profile of 38 patients with DILI and 30 healthy controls found several conjugated molecular species (TCDCA, GCA, TCA, TDCA, and TUDCA) to be elevated, while levels of unconjugated CDCA, DCA, and LCA were lower in patients with DILI compared to healthy controls and higher levels were associated with more severe injury.^10^ These findings likely indicate a hepatic retention of BAs and their spillover into the systemic circulation and suggest the implication of an impaired canalicular BA secretion in the origin of DILI-associated hypercholanemia. In fact, the inhibition of BSEP has been proposed as a contributing cellular mechanism to the development of DILI,^27^ as BA accumulation resulting from BSEP inhibition or deficiency can lead to hepatocyte injury through various mechanisms, including mitochondrial toxicity and the initiation of an inflammatory response.^28^^,^^29^ Many drugs that cause idiosyncratic DILI have been shown to inhibit BSEP activity in vitro,^34^ and polymorphisms in *ABCB11* have been associated with increased risk of developing DILI.^35^ Consistent with this, we found cholestatic type injury was associated with lower levels of secondary BA compared to hepatocellular cases. A study by Quintas et al. (2021), generating discriminant models of DILI subtypes from metabolomic data, reported BAs were one of the main discriminating metabolites of cholestatic DILI, although they were also increased in hepatocellular DILI, albeit to a lesser extent.^36^ Although their study included 79 patients, liver biochemistry threshold to define DILI, its severity and outcomes were not described. In addition, 283 samples were taken from these 79 patients with varying number (1–9) of samples collected from individuals during DILI and the final analysis was enriched with 80 samples from cholestatic DILI compared to 34 hepatocellular DILI. As pattern of liver injury can change during the course of DILI, results from the previous study may not be comparable to our study.

Although significant changes in plasma BAs were observed in patients with DILI as compared to healthy subjects, it was not possible to identify changes specifically attributable to DILI; moreover, BA profiles did not distinguish between DILI and nonDILI cases, restricting utility to prognostication. This observation is not uncommon and limits the use of several proposed new biomarkers as diagnostic tools in DILI.^37^^,^^38^ Our study included 49 cases of nonDILI caused by 4 disorder subtypes, limiting the power of our analysis to identify BA profiles associated with distinct pathologies; varying disruption of the BA pool constituents due to differing injury mechanisms associated with different aetiologies leading to liver dysfunction altering BA metabolism, transport and biosynthesis would be expected. The prospective and longitudinal design of the current study and alignment with the clinical context of acute injury presentation are further strengthened by robust assessment of causality and adjudication of cases. We have assessed BAs at the time of acute injury but the period since drug exposure and relation to peak liver enzyme elevation is variable. Considering that DILI is rare and the majority of cases recover (101/120 in the cohort) without serious consequences such as death or liver transplantation, the statistical power to confirm the ability of BA profiling to pre-empt these endpoints is limited by the small number of events and there is a risk of models overfitting the data especially when adjusting for multiple covariates. However, this is the first prospective cohort study which included consecutive patients with DILI with full range of severity including 19/120 who required transplantation or died. Also since many different drugs, acting via different pathways, can result in DILI, there is heterogeneity in the study groups - this reflects the patients in clinical practice. However, the threshold of the biochemical manifestation to define DILI and classify them into different severity categories is the same for all cases. Further, the study lacks external validation in an independent cohort to assess whether the observed improvement over MELD score is generalisable.

The highly sensitive technique of LC-MS/MS has been used to determine serum levels of total BAs and the most relevant BA species. On the other hand, the complexity and cost of this methodology could restrict large-scale implementation and immediate impact on clinical practice. We have focused on the prognostic implications of serum BAs in DILI involving 120 patients, of which in 28 cases severity progressed, and 19 of them died or required transplantation, two of the most critical outcomes. Incorporating these BA biomarkers enhanced the prognostic performance of MELD, a tool based on traditional blood biomarkers used clinically to indicate prognosis and to prioritise patients for liver transplantation. While MELD alone demonstrated an AUC ROC of 0.76 for identifying progressors and 0.88 for the identification of death or transplantation, these were 0.82 and 0.92, respectively for MELD in combination with the CA + DCA to CDCA + LCA ratio.

In summary, this study has identified two characteristics of the circulating BA profile, i.e., the primary to secondary BAs ratio and the CA + DCA to CDCA + LCA ratio, that can be helpful to enhance the prognostic value of the MELD score and improve early detection of patients where DILI is likely to progress and those with the worst outcomes. Further studies in another independent longitudinal cohort are required to validate this conclusion.

## Contributors

MJM was responsible for data curation, funding acquisition, investigation, methodology, resources, supervision, validation, visualisation and writing the draft manuscript. TDBT was responsible for data curation, formal analysis, methodology, software, visualisation and writing the draft manuscript. JIG was responsible for data curation, funding acquisition, project administration, supervision, visualisation and writing the draft manuscript. DL was responsible for formal analysis, methodology, software, supervision, visualisation and reviewing the manuscript. CS was responsible for data curation, funding acquisition, project administration, and reviewing the manuscript. MIL, RJA, SW, ESB, and AG were responsible for data curation, funding acquisition, project administration, resources, supervision, and reviewing the manuscript. GS and HKB were responsible for data curation, project administration, resources, supervision, and reviewing the manuscript. AKD was responsible for funding acquisition and reviewing the manuscript. AE was responsible for formal analysis and reviewing the manuscript. EA, MRD, WR, JR, AF, WJHG, MEC, JP, AME were responsible for data curation, investigation and reviewing the manuscript. ML was responsible for data curation, project administration and reviewing the manuscript. OP was responsible for funding acquisition, data curation, methodology, project administration, resources, validation, and reviewing the manuscript. SKR was responsible for funding acquisition, project administration, and reviewing the manuscript. SLS was responsible for data curation, project administration, resources, and reviewing the manuscript. SAP was responsible for funding acquisition, project administration, data curation and reviewing the manuscript. JJGM was responsible for funding acquisition, investigation, methodology and reviewing the manuscript. GAKU was responsible for conceptualisation, funding acquisition, project administration, resources, and reviewing the manuscript. GPA was responsible for conceptualisation, funding acquisition, project administration, resources, and writing the draft manuscript. MJM, TDBT and JIG contributed equally and are joint first authors. GAKU and GPA contributed equally and are joint senior authors. All authors read and approved the final version of the manuscript. MJM, TDBT, DL, JIG, GPA accessed and verified the underlying data.

## Data sharing statement

The bile acid levels determined in the study participants are available in Mendeley (https://doi.org/10.17632/sf6w9yyvnj.1). The participant data that support the findings of this study (in deidentified format) are available for medical research purposes on written request to the corresponding author with provision of a data transfer agreement with the source institutions, upon publication.

## Declaration of interests

Oliver Poetz is shareholder of SIGNATOPE GmbH. Gerd A. Kullak-Ublick holds Novartis and TransHeps AG equity. Dingzhou Li, Thi Dong-Binh Tran, Sara Paciga, Shashi K. Ramaiah were employed by Pfizer when the study was performed. Guruprasad P. Aithal has received consulting fees from Amryth, Agios, DNDi, PureTech LYT Inc, Pfizer Inc, GlaxoSmithKline, Clinipace, Merck Healthcare KGaA, JnJ, Suzhou MDCE Co Ltd, SynOx Therapies, Novartis Pharma, AstraZeneca and BenevolentAI Bio, paid to the University of Nottingham and has received investigator-led research funding from Ipsen Ltd and Pfizer Inc. John Ramage has received speaker fees from Ipsen Ltd. William Griffiths received payment from EASL, BASL, BSG, RCPE as a speaker and as advisory board member for Health Advances. Edmond Atallah received payment from Dr Falk and BMS for lectures. All other authors have no conflicts to declare.

## References

[1] Ferrell J.M., Chiang J.Y.L. (2021). Bile acid receptors and signaling crosstalk in the liver, gut and brain. Liver Res.

[2] Trauner M., Boyer J.L. (2003). Bile salt transporters: molecular characterization, function, and regulation. Physiol Rev.

[3] Hofmann A.F. (2009). The enterohepatic circulation of bile acids in mammals: form and functions. Front Biosci.

[4] Hagenbuch B., Stieger B. (2013). The SLCO (former SLC21) superfamily of transporters. Mol Asp Med.

[5] Aithal G., Kullak-Ublick G.A., Arias I.M.A.H., Boyer J.L., Cohen D.E., Shafritz D.A., Thorgeirsson S.S., Wolkoff A.W. (2020). The Liver Biology and Pathobiology.

[6] Koido M., Kawakami E., Fukumura J. (2020). Polygenic architecture informs potential vulnerability to drug-induced liver injury. Nat Med.

[7] Nunes de Paiva M.J., Pereira Bastos de Siqueira M.E. (2005). Increased serum bile acids as a possible biomarker of hepatotoxicity in Brazilian workers exposed to solvents in car repainting shops. Biomarkers.

[8] Woolbright B.L., McGill M.R., Staggs V.S. (2014). Glycodeoxycholic acid levels as prognostic biomarker in acetaminophen-induced acute liver failure patients. Toxicol Sci.

[9] Luo L., Aubrecht J., Li D. (2018). Assessment of serum bile acid profiles as biomarkers of liver injury and liver disease in humans. PloS One.

[10] Ma Z., Wang X., Yin P. (2019). Serum metabolome and targeted bile acid profiling reveals potential novel biomarkers for drug-induced liver injury. Medicine (Baltimore).

[11] Xie Z., Zhang L., Chen E. (2021). Targeted metabolomics analysis of bile acids in patients with idiosyncratic drug-induced liver injury. Metabolites.

[12] Yu S., Wang S., Li P. (2025). Integrated analysis of serum and fecal metabolites reveals the role of bile acid metabolism in drug-induced liver injury: implications for diagnostic and prognostic biomarkers. J Clin Transl Hepatol.

[13] Azer S.A., Hasanato R. (2021). Use of bile acids as potential markers of liver dysfunction in humans: a systematic review. Medicine (Baltimore).

[14] Atallah E., Freixo C., Alvarez-Alvarez I. (2021). Biomarkers of idiosyncratic drug-induced liver injury (DILI) - a systematic review. Expert Opin Drug Metab Toxicol.

[15] Andrade R.J., Chalasani N., Björnsson E.S. (2019). Drug-induced liver injury. Nat Rev Dis Primers.

[16] Church R.J., Kullak-Ublick G.A., Aubrecht J. (2019). Candidate biomarkers for the diagnosis and prognosis of drug-induced liver injury: an international collaborative effort. Hepatol.

[17] Grove J.I., Stephens C., Lucena M.I. (2023). Study design for development of novel safety biomarkers of drug-induced liver injury by the translational safety biomarker pipeline (TransBioLine) consortium: a study protocol for a nested case–control study. Diagn Progn Res.

[18] Aithal G.P., Watkins P.B., Andrade R.J. (2011). Case definition and phenotype standardization in drug-induced liver injury. Clin Pharmacol Ther.

[19] (2019). EASL Clinical Practice Guidelines: Drug-induced liver injury. J Hepatol.

[20] Uriarte I., Santamaria E., López-Pascual A. (2024). New insights into the regulation of bile acids synthesis during the early stages of liver regeneration: a human and experimental study. Biochim Biophys Acta Mol Basis Dis.

[21] Ye L., Liu S., Wang M., Shao Y., Ding M. (2007). High-performance liquid chromatography-tandem mass spectrometry for the analysis of bile acid profiles in serum of women with intrahepatic cholestasis of pregnancy. J Chromatogr B Analytl Technol Biomedical Life Sci.

[22] Vickers A.J., van Calster B., Steyerberg E.W. (2019). A simple, step-by-step guide to interpreting decision curve analysis. Diagn Progn Res.

[23] Tao W., Fan Q., Wei J. (2024). Gut-Liver Axis as a therapeutic target for drug-induced liver injury. Curr Issues Mol Biol.

[24] Alamoudi J.A., Li W., Gautam N. (2021). Bile acid indices as biomarkers for liver diseases I: diagnostic markers. World J Hepatol.

[25] Jahan A., Chiang J.Y. (2005). Cytokine regulation of human sterol 12alpha-hydroxylase (CYP8B1) gene. Am J Physiol Gastrointest Liver Physiol.

[26] Zhao S., Fu H., Zhou T. (2022). Alteration of bile acids and omega-6 PUFAs are correlated with the progression and prognosis of drug-induced liver injury. Front Immunol.

[27] Kenna J.G., Taskar K.S., Battista C. (2018). Can bile salt export pump inhibition testing in drug discovery and development reduce liver injury risk? An international transporter consortium perspective. Clinl Pharmacol Ther.

[28] Perez M.J., Briz O. (2009). Bile-acid-induced cell injury and protection. World J Gastroenterol.

[29] Kullak-Ublick G.A., Andrade R.J., Merz M. (2017). Drug-induced liver injury: recent advances in diagnosis and risk assessment. Gut.

[30] Zhang M., Chiang J.Y. (2001). Transcriptional regulation of the human sterol 12alpha-hydroxylase gene (CYP8B1): roles of heaptocyte nuclear factor 4alpha in mediating bile acid repression. J Biol Chem.

[31] Ovadia C., Seed P.T., Sklavounos A. (2019). Association of adverse perinatal outcomes of intrahepatic cholestasis of pregnancy with biochemical markers: results of aggregate and individual patient data meta-analyses. Lancet.

[32] Thompson R.J., Arnell H., Artan R. (2022). Odevixibat treatment in progressive familial intrahepatic cholestasis: a randomised, placebo-controlled, phase 3 trial. Lancet Gastroenterol Hepatol.

[33] Mireault M., Rose C.F., Karvellas C.J., Sleno L. (2024). Perturbations in human bile acid profiles following drug-induced liver injury investigated using semitargeted high-resolution mass spectrometry. Rapid Commun Mass Spectrom.

[34] Dawson S., Stahl S., Paul N., Barber J., Kenna J.G. (2012). In vitro inhibition of the bile salt export pump correlates with risk of cholestatic drug-induced liver injury in humans. Drug Metab Dispos.

[35] Ulzurrun E., Stephens C., Crespo E. (2013). Role of chemical structures and the 1331T>C bile salt export pump polymorphism in idiosyncratic drug-induced liver injury. Liver Int.

[36] Quintás G., Martínez-Sena T., Conde I., Pareja Ibars E., Kleinjans J., Castell J.V. (2021). Metabolomic analysis to discriminate drug-induced liver injury (DILI) phenotypes. Arch Toxicol.

[37] Llewellyn H.P., Vaidya V.S., Wang Z. (2021). Evaluating the sensitivity and specificity of promising circulating biomarkers to diagnose liver injury in humans. Toxicol Sci.

[38] Ravindra K.C., Vaidya V.S., Wang Z. (2023). Tandem mass tag-based quantitative proteomic profiling identifies candidate serum biomarkers of drug-induced liver injury in humans. Nat Commun.

