# Supplementary material for: Usefulness of plasma bile acid profile as a prognostic biomarker for drug-induced liver injury
Source: eBioMedicine. 2026 Mar 24;126:106229. doi: 10.1016/j.ebiom.2026.106229 (PMC13049577; doi:10.1016/j.ebiom.2026.106229)
Supplement: Supplementary Materials [file mmc1.pdf]

Usefulness of plasma bile acid profile as a prognostic biomarker for drug-induced liver injury

Monte, MJ., Tran, DBH., Grove JI. et al. 2026

Contents **Page**

Supplemental Methods [pg 1-3]:
Study Design 1
Sample Size 1
Biomarker Analysis 2
Statistical Analysis 3

Supplemental Tables [pg 4-12]:
Table S1. Recruiting centres and ethical approvals. 4
Table S2. Recruitment eligibility criteria and description of disease causes for each cohort. 5
Table S3. Analysis of Inter-centre variability at the 3 main recruiting centres. 6
Table S4. Bile acid profiles for study groups. 7
Table S5. Bile acid profiles for DILI sub-groups. 8
Table S6. FDR corrected p values from ANOVA. 9
Table S7. Univariate analysis to identify biomarkers with performance above AUC 0.7 for death/transplantation outcome in DILI patients. 10
Table S8. Univariate analysis to identify biomarkers with performance above AUC 0.7 for disease progression in DILI patients. 10
Table S9. Bivariate analysis to identify bile acid biomarkers which when combined with MELD have performance above AUC 0.75 for disease progression in DILI patients (n=114). 11
Table S10. Bivariate analysis to identify best performing bile acid biomarkers which when combined with MELD have performance ≥AUC 0.88 for death/transplantation outcomes in DILI patients (n=116). 12

Supplemental Figures [pg 13-17]:
Figure S1. Levels of selected bile acids in patients with acute nonDILI stratified according to liver injury severity. 13
Figure S2. Levels of selected bile acids in patients with acute DILI stratified according to liver injury type. 14
Figure S3. Levels of selected bile acids in patients with acute nonDILI stratified according to liver injury type. 15
Figure S4. Decision Curve Analysis for Progression. 16
Figure S5. Decision Curve Analysis for Outcomes of death/liver transplantation. 17

Supplemental References [pg 18].

Supplemental Methods

Study Design

A nested case-control observational study design has been devised to prospectively identify and enroll a cohort of patients with acute liver injury at presentation at secondary care centres in six European countries through attendance for standard clinical care pathways from 6/9/19 to 27/3/24.[1] Patients are assessed clinically and through investigations (laboratory tests and imaging) and sub-grouped as DILI ('cases') or acute 'nonDILI' ('controls'). Both cases and controls are followed up to recovery or death or transplantation. Blood samples are obtained from both groups at presentation. In addition, samples from healthy volunteers (HV) were collected in Belgium for comparison from 18/3/21 to 7/12/21. Ethical approvals were obtained from local Ethical Review Authorities for each of the recruiting locations (Table S1). The inclusion and exclusion criteria for each study cohort are detailed in Table S2.

Sample Size

For using the Ratio of (CA+DCA)/(CDCA+LCA) as a prognostic biomarker, at the statistical power of 85% and the significance level of 5%, the number of progressors/non progressors needed is 25/77, while number of death/transplantation or non-death/transplantation cases needed is 16/81. Our cohort have an adequate sample size with 28 progressors and 90 non-progressors, and 19 death/transplantation and 101 non-death/transplantation.

Biomarker Analysis

EDTA-blood samples were collected at the study visit and separated by centrifugation and stored at -80ºC until analysis. As outlined in the study analysis plan, a sub-set of DILI cases for analysis (n=120) were selected from those available at date of bile acid (BA) analysis in the prospectively-recruited TransBioLine study DILI cohort,[1] (n=217) at random (randomisation.com), but with inclusion of all available cases with death/transplantation outcome or progression in severity. For nonDILI cases, 49/85 were randomly selected. HV samples were selected following prospective recruitment as: all 15 available at time of first batch of bile acid (BA) analysis in June 2022 and then a further 10 randomly selected from those available in Sept 2023. Analysis was completed at the University of Salamanca in batches during the project (June 2022: 13 DILI and 27 nonDILI; Sept 2022: 15 HV and 25 DILI; Sept 2023: 10 HV, 47 DILI and 23 nonDILI; Jul 2024: 41 DILI).

BAs are abbreviated as: αMCA, Alpha-Muricholic acid; βMCA, Beta-Muricholic acid; CA, Cholic acid; CDCA, Chenodeoxycholic acid; DCA, Deoxycholic acid; GUDCA, Glycoursodeoxycholic acid; GCA, Glycocholic acid; GCDCA, Glycochenodeoxycholic acid; GDCA, Glycodeoxycholic acid; GLCA, Glycolithocholic acid; HyoDCA, Hyodeoxycholic acid; LCA, Lithocholic acid; Ratio12αOH/non12αOH, Ratio of 12alpha-hydroxylated to non-12alpha hydroxylated bile acids; SLCA, Sulfolithocholic acid; TCA, Taurocholic acid; TCDCA, Taurochenodeoxycholic acid; TDCA, Taurodeoxycholic acid; THCA,Trihydroxycholestanoic acid TLCA, Taurolithocholic acid; TMCAs, Tauromuricholic acids; TSLCA, Taurosulfolithocholic acid; TUDCA, Tauroursodeoxycholic acid; UDCA, Ursodeoxycholic acid.

BAs used as standards in the analysis were purchased from the following distributors: CA, CDCA, DCA, LCA,UDCA, αMCA and βMCA) and, as well as their tauroconjugated and glycoconjugated forms, HyoDCA, SLCA and TSLCA were from Sigma-Aldrich (Madrid, Spain). THCA was from Instruchemie (Groningen, The Netherlands). Nor-deoxycholic acid (Nor-DCA) used as internal standard was from Toronto Research Chemicals (Toronto, Canada). According to the suppliers the purity of these compounds was ≥97%.

BAs were extracted from EDTA-plasma samples by using silica-based bonded phase cartridges (Sep-Pack Plus C18, Waters, Madrid).[2] Then, chromatographic separation was achieved with gradient elution using a Zorbax Eclipse XDB-C18 column (150 mm x 4.6 mm, 5 µm) kept at 35ºC and a flow rate of 500 µl/min. Initial mobile phase was 73:27 methanol/water, both containing 5 mM ammonium acetate and 0.01% formic acid, pH 4.6, and it was changed to 97:3 methanol/water over 10 min and then returned to 73:27 for 1 min. Electrospray ionization (ESI) in negative mode was used, with the following conditions: gas temperature 350ºC, gas flow 11 l/min, nebulizer 45 psi, capillary voltage 2500 V. MS/MS acquisition was performed in multiple reaction monitoring (MRM) mode using the specific *m/z* transitions: $[M-H]^-$ ion to 80.2 for taurine-conjugated BAs and $[M-H]^-$ ion to 74 for glycine-conjugated BAs. As reported by others, free BAs do not generate characteristic ion fragments.[3] Transition from unfragmented precursor molecular ions 407.1 to 407.1, 391.3 to 391.3 and 375.3 to 375.3 were selected for trihydroxylated, dihydroxylated and monohydroxylated unconjugated BAs, respectively. Transition 377.0 to 331.3 *m/z* was followed for the internal standard nor-DCA. The recovery of the whole analytical process, which included extraction from serum, followed by chromatographic separation using HPLC and quantification by MS/MS, was determined by the measurement of the added internal standard, i.e., Nor-DCA. Mean recovery (±SEM) for samples in the DILI group (n=120) was 89.0±0.8%, which was very close to that for the nonDILI group (n=49; 87.0±1.6%). The accuracy of calibration curves was calculated at an intermediate point of the concentration range, i.e., 5 µM. The results indicated that the average value (±SEM) was 91.6±0.8% for 230 calibration curves, with no significant differences among bile acid species. Reproducibility was calculated at two concentrations of the calibration curve for each of the 22 BAs analyzed. The coefficients of variation (CVs) were lower than 15% (range 2.8-14.5%) at 1 µM, and lower than 2% (range 0.8-1.8%) at 25 µM.

Plasma concentrations of 22 molecular BA species were analysed, and these values were used to calculate levels of total BAs, primary BAs, secondary BAs, and major BA families, as well as some pathophysiologically significant ratios, e.g., the ratio of primary/secondary BAs, glycoconjugated/tauroconjugated BAs, etc.

Biomarkers alanine aminotransferase, ALT, (U/L), total bilirubin, TBIL, (mg/dL), creatinine (mg/dL) and albumin (g/L), were quantified in serum samples from healthy volunteers using standard clinical biochemistry assays (Cobas 6000 system, Roche) at MLM Medical Labs. For analysis, healthy volunteer measurements of albumin were converted to g/dL to align to clinical reporting for comparison across cohorts. Values lower than the lower limits of quantification (LLOQ) were imputed by the LLOQ value. For patient cohorts ALT (U/L), ALP (U/L), TBIL (mg/dL), albumin (g/dL), creatinine (mg/dL) and INR were clinically-determined. MELD was calculated by = 3.78*log(TBIL) + (11.2*log(INR)) + (9.57*log(Creatinine)) + 6.43) as specified by Kamath and Kim, 2007;[4] TBIL and creatinine is in mg/dL. The lower limit for serum creatinine, serum TBIL, and INR

separate components was fixed at 1 (each) so that there would be no negative scores; the upper limit of serum creatinine was capped at 4 mg/dL.

Statistical Analysis

It was hypothesized that the BA concentration and protein biomarker are a function of clinical diagnosis (DILI, nonDILI and healthy volunteers), confounded by demographic factors, such as gender, age and BMI.

For statistical analysis log transformed values of concentration were used. Inter-centre variation was assessed across the 3 main recruiting sites (Table S3). The 40 BA biomarkers measured are metabolites from biological processing having inter-dependency. To determine the prognostic performance for each BA and candidate protein biomarker in both DILI progression and death/transplantation outcome of DILI, receiver operator characteristic (ROC) curve analyses were utilized (Table S7 and S8). The ROC area under the curve (AUC) offers an estimator of the overall behaviour of all data points along the path of the curve and accuracy of a test by calculating simultaneously the sensitivity and specificity.[5] It is the probability of ranking a randomly selected individual with an event over a randomly selected individual without the event. The 95% confidence intervals for AUC were estimated using the 'DeLong' method. Sensitivity is the proportion of actual progressor patients or death/transplantation cases that were correctly identified by the model and equals to True Positives / (True Positives + False Negatives), while specificity is the proportion of actual non-progressor patients or no-death/transplantation cases that were correctly identified and calculated by True Negatives / (True Negatives + False Positives). FDR correction of p values reported in Table 3 and Table S5 is provided in Table S6.

Bivariate logistic regression was also performed for both DILI progression (28 progressors vs. 90 no-progressors; 2 cases had unknown progression) and DILI severity outcome (19 cases with death/transplant versus 101 cases without death/transplant) as above to identify the best prognostic ability of any pair of BAs or any pair of protein biomarkers. An AUC of 0.75 or higher was considered as acceptable discrimination. Bivariate logistic regression shows better prediction performance by considering synergy of both biomarkers and consequently the AUC value is higher than the one in the univariate logistic regression model. Lower residual deviance was considered as an indicator of model best fit.

Integrated discrimination improvements (IDI) were proposed as an alternative to the increase in AUC for evaluating improvement in the performance of risk assessment introduced by the addition of new biomarker for BA data (Table S9 and S10). IDI is a tool for evaluating capacity of a biomarker to predict a binary outcome of interest by summarizing the extent a new model increases risk in events and decreases risk in non-events. [6,7] The range of IDI is -1 to 1. The higher the IDI, the larger the average predicted risks between DILI patients who did progress or had death/transplantation outcome versus patients who did not, when adding a new biomarker.

Decision Curve Analysis (Fig S4 and S5) is an approach of estimating clinical "net benefit" for one or several prediction models or prognostic/diagnostic tests in comparison to baseline models of treating/intervention all or no patients. We had calculated the net benefit as the minimum probability of DILI progression or DILI death/transplantation cases at which further prognostic/diagnostic tests for BA would be warranted. The net benefit is computed across a range of threshold probabilities and equals to (True Positives / N) – (False Positives / N) x pt(1 - pt), where N is the number of observations, and pt is the threshold probability. Net benefit goes beyond the traditional accuracy and explicitly uses patient/clinician risk tolerance for guiding clinical decision. The highest net benefit curve across relevant threshold indicates the best model for clinical utility.[8]

All analyses were performed using R version 4.3.2.

Supplemental Tables

Table S1. Recruiting centres and ethical approvals.

Centre	Location	Ethical Approval
Addenbrookes University Hospital, Cambridge University Hospitals NHS Trust Hospital	Cambridge, UK	UK Health Research Authority (Refs: 15/YH/0294; GM010201; 14/EM/0145)
Hampshire Hospitals NHS Foundation Trust,	Basingstoke, UK	
Nottingham University Hospitals NHS Trust	Nottingham, UK	
Derriford Hospital, University Hospitals Plymouth NHS Trust	Plymouth, UK	
Queen Alexandra Hospital, Portsmouth Hospitals NHS Trust	Portsmouth, UK	
Royal Cornwall Hospitals NHS Trust	Treliske, UK	
Royal Free Hospital	London, UK	
University Hospitals Birmingham	Birmingham, UK	
University Hospitals Trust Southampton	Southampton, UK	
Lanspitali University Hospital	Reykjavik, Iceland	Bioethics Committee Iceland (Ref: 15-104-V1)
Malaga University Hospital	Malaga, Spain	Biomedical Investigation Ethics Committee of Andalucia (Ref: AND-HEP-2015-01)
Munich University Hospital	Munich, Germany	Ethical Commission of Ludwig Maximilian University of Munich (Project 85-16)
Sahlgrenska University Hospital	Gothenburg, Sweden	Swedish Ethics Review Authority (Ref: 2022-04078-01)
University Hospital Bern	Bern, Switzerland	Bern Cantonal Ethics Committee for Research (Ref: 2016-00932)
University Hospital Zurich	Zurich, Switzerland	Zurich Cantonal Ethics Committee (Ref. 2019-01451)
Pfizer Global Research & Development (recruiting healthy volunteers only)	Brussels, Belgium	Ethics Committee Erasme Hospital (Ref: P2019/530).

Table S2. Recruitment eligibility criteria and description of disease causes for each cohort.

**Cohorts**	**Drug-induced liver injury (DILI) n=120**	**Other acute liver injury (nonDILI) n=49**	**Healthy volunteers (HV) n=25**
**Inclusion criteria**	▪ Age ≥18<br>▪ Exposure to potential causal agent<br>▪ Meets one of the following analytical thresholds at enrolment (visit 1):<br>- ALT ≥5x ULN<br>- ALP ≥2x ULN<br>- ALT ≥3x ULN + TBIL >2x ULN<br>▪ Diagnosis of acute DILI confirmed by panel	▪ Age ≥18<br>▪ Exposure to potential causal agent<br>▪ Meets one of the following analytical thresholds at enrolment (visit 1):<br>- ALT ≥5x ULN<br>- ALP ≥2x ULN<br>- ALT ≥3x ULN +TBIL >2x ULN<br>▪ Diagnosis of nonDILI (alternate cause) acute liver injury by panel	▪ Age ≥18<br>▪ BMI ≤32<br>▪ Normal liver enzymes
**Exclusion criteria**	▪ Clear diagnosis of alternative acute liver condition (e.g. acute viral or autoimmune hepatitis unrelated to the drug)	▪ Relapse of previously diagnosed chronic liver injury	▪ Diagnosis of liver disease<br>▪ Transient Elastography CAP score of ≥260 dB/m
**Injury type**[9]	▪ Hepatocellular: n=77<br>▪ Cholestatic: n=21<br>▪ Mixed type: n=22	▪ Hepatocellular: n=34<br>▪ Cholestatic: n=6<br>▪ Mixed type: n=9	▪ n/a
**Determined cause of injury**	▪ Suspected drugs (1 case or number in parentheses): Abbiraterone, Albendazole, Alverine, Amoxicillin-clavulanate (7), Atorvastatin (8), Azathioprine, Cemiplimab, Deferasirox, Disulfiram (2), Doxycycline (2), Flucloxacillin (7), herbal products (5), Ibuprofen, Infliximab (5), Inotuzumab, Ipilumamab-Nivolumab (16), Isoniazid (2), Itraconazole, Lamotrigine, Leflunomide, Levetiracetam, Metamizole, Methylprenisolone, Nitrofurantoin (4), Nivolumab (2), Pazopanib, Pembrolizumab (5), Piperacillin-Tazobactam, Ribocilib, Sulfamethoxazole-Trimethoprim, Thiamazole, Tibolone, Trimipramine, Vedolizumab, Zanubrutinib, polypharmacy (32)	▪ Autoimmune hepatitis: n=22<br>▪ Biliary obstruction n=12<br>▪ Viral hepatitis: n=10<br>▪ Other (e.g. ischemic hepatitis, pancreatic cancer) n=5	▪ n/a

ALP, alkaline phosphatase; ALT, alanine aminotransferase; CAP Controlled attenuation parameter; ULN, upper limit of normal; TBIL, total bilirubin.

Table S3. Analysis of Inter-centre variability at the 3 main recruiting centres (Spain, Germany, UK).

Variable	Centre 1	Centre 2	Centre 3	Fisher's exact test p value
**Total number recruited**	31	31	71	
**Cohort; n (%)**				
DILI	16 (52%)	23 (74%)	48 (68%)	0.2
NonDILI	15 (48%)	8 (26%)	23 (32%)	
**Severity; n (%)**				
mild	8 (50%)	7 (30%)	24 (50%)	0.2
moderate	8 (50%)	10 (43%)	18 (38%)	
severe	0 (0%)	6 (26%)	6 (13%)	
**Progression in severity; n (%)**	2 (13%)	6 (27%)	6 (13%)	0.7
**Outcome of death or liver transplant; n (%)**	1 (6.3%)	3 (13%)	11 (23%)	0.3

Table S4. Bile acid profiles for study groups.

**Bile Acids µM** Mean±SD (Min-Max)	**Cohort**			**DILI severity sub-groups**		
	**HV** n = 25	**NonDILI** n = 49	**DILI** n = 120	**Mild** n = 56	**Moderate** n = 46	**Severe** n = 18
GCA	0.08±0.07 (0.02-0.32)	36.32±40.09 (0.07-179.12)[a]	30.51±43.39 (0.06-259.84)[a]	7.26±20.56 (0.06-110.59)	49.18±52.92 (0.73-259.84)[c]	55.11±32.15 (0.32-130.83)[c]
GCDCA	0.27±0.24 (0.05-0.89)	22.96±23.12 (0.22-91.01)[a]	22.16±35.06 (0.15-239.21)[a]	5.29±12.42 (0.15-58.45)	24.08±27.81 (0.44-104.23)[c]	69.72±52.09 (0.25-239.21)[c,d]
GDCA	0.15±0.13 (0.00-0.51)	1.10±4.74 (0.00-33.31)	1.13±3.37 (0.00-29.45)	1.15±4.01 (0.00-29.45)	1.00±2.60 (0.00-14.27)	1.38±3.09 (0.00-11.99)
GLCA	0.01±0.01 (0.00-0.03)	0.02±0.02 (0.00-0.12)	0.02±0.03 (0.00-0.16)	0.02±0.03 (0.00-0.16)	0.02±0.03 (0.00-0.14)	0.04±0.04 (0.00-0.14)
GUDCA	0.03±0.04 (0.00-0.15)	0.20±0.32 (0.00-1.47)[a]	0.33±1.45 (0.00-15.77)[a]	0.40±2.10 (0.01-15.77)	0.22±0.33 (0.00-1.39)	0.42±0.42 (0.01-1.40)[c]
TCA	0.01±0.01 (0.00-0.07)	18.48±19.11 (0.02-73.68)[a]	21.32±36.26 (0.01-206.85)[a]	3.84±10.25 (0.01-58.90)	41.66±49.02 (0.18-206.85)[c]	23.68±19.27 (0.07-73.35)[c]
TCDCA	0.03±0.03 (0.01-0.14)	12.96±11.63 (0.05-44.18)[a]	12.90±20.48 (0.02-140.70)[a]	2.85±7.68 (0.02-44.64)	17.36±19.65 (0.26-86.69)[c]	32.78±30.29 (0.23-140.70)[c]
TDCA	0.02±0.02 (0.00-0.11)	0.78±2.26 (0.00-15.27)[a]	0.77±1.81 (0.00-11.02)[a]	0.48±1.46 (0.00-9.47)	1.09±2.31 (0.00-11.02)	0.82±1.18 (0.00-4.75)
TLCA	0.001±0.001 (0.00-0.003)	0.011±0.017 (0.00-0.093)[a]	0.011±0.016 (0.00-0.078)[a]	0.008±0.011 (0.000-0.059)	0.013±0.017 (0.000-0.075)	0.020±0.021 (0.000-0.078)
TUDCA	0.002±0.002 (0.000-0.009)	0.090±0.155 (0.00-0.869)[a]	0.121±0.587 (0.00-6.366)[a]	0.13±0.85 (0.00-6.37)	0.09±0.14 (0.00-0.80)[c]	0.15±0.16 (0.01-0.65)[c]
TSLCA	0.08±0.08 (0.01-0.40)	0.31±0.32 (0.03-1.73)[a]	0.56±0.71 (0.00-3.13)[a]	0.35±0.48 (0.00-2.43)	0.93±0.89 (0.02-3.13)[c]	0.24±0.23 (0.03-0.88)
TMCA	0.005±0.005 (0.000-0.017)	0.202±0.293 (0.002-1.393)[a]	0.154±0.305 (0.000-2.705)[a]	0.093±0.181 (0.000-0.821)	0.255±0.431 (0.009-2.705)[c]	0.089±0.111 (0.001-0.477)
CA	0.19±0.22 (0.02-0.91)	0.52±1.71 (0.01-11.47)	0.33±0.64 (0.02-4.72)	0.24±0.44 (0.03-2.75)	0.48±0.88 (0.03-4.72)	0.24±0.25 (0.02-1.06)
CDCA	0.09±0.13 (0.01-0.65)	0.12±0.39 (0.00-2.68)	0.13±0.27 (0.00-2.12)	0.13±0.30 (0.00-2.12)	0.09±0.22 (0.00-1.08)	0.21±0.28 (0.01-1.05)[d]
DCA	0.21±0.16 (0.01-0.64)	0.07±0.17 (0.00-0.85)[a]	0.09±0.16 (0.00-0.88)[a]	0.17±0.20 (0.00-0.88)	0.03±0.06 (0.00-0.33)[c]	0.01±0.02 (0.00-0.06)[c]
LCA	0.010±0.005 (0.002-0.021)	0.006±0.006 (0.00-0.024)[a]	0.009±0.012 (0.00-0.096)[a]	0.012±0.016 (0.000-0.096)	0.005±0.006 (0.000-0.034)[c]	0.006±0.007 (0.000-0.024)
UDCA	0.018±0.018 (0.003-0.085)	0.013±0.045 (0.00-0.308)[a]	**0.017±0.041** (0.00-0.388)[b]	0.027±0.057 (0.001-0.388)	0.009±0.012 (0.000-0.051)[c]	0.006±0.006 (0.001-0.021)
SLCA	0.004±0.002 (0.000-0.010)	0.006±0.007 (0.000-0.027)	0.012±0.018 (0.000-0.124)	0.009±0.016 (0.000-0.083)	0.010±0.012 (0.000-0.055)	0.023±0.031 (0.000-0.124)
αMCA	0.006±0.004 (0.001-0.018)	0.003±0.002 (0.000-0.010)[a]	**0.004±0.004** (0.000-0.023)[b]	0.004±0.004 (0.000-0.022)	0.005±0.004 (0.001-0.023)	0.002±0.002 (0.000-0.008)
βMCA	0.003±0.003 (0.001-0.013)	0.004±0.009 (0.000-0.053)	0.003±0.003 (0.000-0.025)	0.002±0.002 (0.000-0.012)	0.003±0.004 (0.000-0.025)	0.004±0.004 (0.000-0.014)
HyoDCA	0.030±0.022 (0.002-0.086)	0.011±0.018 (0.001-0.078)[a]	0.020±0.029 (0.000-0.154)[a]	0.026±0.031 (0.000-0.141)	0.017±0.031 (0.000-0.154)	0.011±0.008 (0.001-0.032)
THCA	0.01±0.00 (0.00-0.02)	0.01±0.01 (0.00-0.06)[a]	0.01±0.01 (0.00-0.07)[a]	0.01±0.01 (0.00-0.07)	0.02±0.01 (0.00-0.04)[c]	0.02±0.01 (0.01-0.04)[c]
CA Family	0.28±0.23 (0.05-0.98)	55.32±56.18 (0.20-205.36)[a]	52.16±76.01 (0.12-462.86)[a]	11.34±29.26 (0.12-139.21)	91.32±96.64 (1.38-462.86)[c]	79.04±50.48 (0.41-195.59)[c]
CDCA Family	0.39±0.35 (0.07-1.56)	36.04±33.85 (0.39-123.07)[a]	35.19±54.14 (0.18-380.70)[a]	8.27±19.53 (0.18-103.14)	41.53±44.65 (1.12-161.94)[c]	102.71±81.75 (0.49-380.70)[c]
DCA Family	0.37±0.28 (0.01-1.10)	1.95±6.98 (0.00-48.86)	1.99±4.93 (0.00-39.07)	1.80±5.38 (0.01-39.07)	2.12±4.74 (0.00-22.29)	2.21±4.08 (0.00-14.51)
LCA Family	0.10±0.08 (0.01-0.43)	0.35±0.35 (0.04-1.99)[a]	0.61±0.73 (0.00-3.20)[a]	0.40±0.51 (0.00-2.50)	0.98±0.91 (0.02-3.20)[c]	0.33±0.27 (0.03-1.05)
UDCA Family	0.05±0.05 (0.01-0.19)	0.30±0.44 (0.00-2.07)[a]	0.47±2.07 (0.01-22.52)[a]	0.56±3.00 (0.01-22.52)	0.32±0.45 (0.01-2.20)	0.58±0.57 (0.02-2.07)[c]

Tukey adjusted p value (grey fill indicates significance): [a]nonDILI or DILI group is significantly different from HV group; [b]DILI group is significantly different from nonDILI group (value shown in bold); [c]Moderate or Severe group is significantly different from Mild group; [d]Severe group is significantly different from Moderate group.

Table S5. Bile acid profiles for DILI sub-groups.

	DILI: Progression Group		DILI: Outcome Death/ Liver Transplantation	
**Bile Acids µM** Mean±SD (Min-Max)	**No Progression** n = 90	**Progressed** n = 28	**No** n = 101	**Yes** n = 19
GCA	30.47±47.00 (0.06-259.84)	32.71±30.81 (0.32-105.01)	29.04±45.50 (0.06-259.84)	38.32±29.48 (0.32-105.01)[a]
GCDCA	17.67±27.70 (0.15-104.23)	38.05±50.25 (0.25-239.21)[a]	16.90±26.68 (0.15-104.23)	50.08±56.49 (0.25-239.21)[a]
GDCA	1.19±3.75 (0.00-29.45)	1.00±1.89 (0.00-7.44)	1.19±3.61 (0.00-29.45)	0.80±1.60 (0.00-6.85)
GLCA	0.02±0.03 (0.00-0.16)	0.03±0.03 (0.00-0.14)	0.02±0.03 (0.00-0.16)	0.03±0.04 (0.00-0.14)
GUDCA	0.37±1.67 (0.00-15.77)	0.25±0.32 (0.01-1.40)	0.34±1.58 (0.00-15.77)	0.31±0.35 (0.01-1.40)
TCA	22.61±39.99 (0.01-206.85)	18.64±21.91 (0.07-75.17)	21.25±38.36 (0.01-206.85)	21.66±22.71 (0.07-75.17)
TCDCA	11.13±17.83 (0.02-86.69)	19.48±27.09 (0.16-140.70)[a]	10.54±17.15 (0.02-86.69)	25.46±30.67 (0.17-140.70)[a]
TDCA	0.77±1.96 (0.00-11.02)	0.80±1.33 (0.00-4.75)	0.78±1.91 (0.00-11.02)	0.67±1.13 (0.00-4.75)
TLCA	0.010±0.014 (0.000-0.075)	0.017±0.020 (0.000-0.078)	0.011±0.014 (0.000-0.075)	0.016±0.021 (0.000-0.078)
TUDCA	0.13±0.67 (0.00-6.37)	0.10±0.14 (0.00-0.65)[a]	0.12±0.64 (0.00-6.37)	0.14±0.16 (0.01-0.65)[a]
TSLCA	0.54±0.70 (0.01-3.01)	0.65±0.75 (0.03-3.13)	0.57±0.71 (0.00-3.01)	0.50±0.73 (0.03-3.13)
TMCA	0.168±0.340 (0.000-2.705)	0.120±0.164 (0.001-0.716)	0.171±0.329 (0.000-2.705)	0.068±0.070 (0.001-0.272)
CA	0.36±0.71 (0.03-4.72)	0.25±0.35 (0.02-1.86)	0.36±0.69 (0.03-4.72)	0.16±0.15 (0.02-0.56)
CDCA	0.11±0.27 (0.00-2.12)	0.18±0.24 (0.01-1.05)	0.11±0.26 (0.00-2.12)	0.21±0.28 (0.01-1.05)[a]
DCA	0.11±0.18 (0.00-0.88)	0.04±0.05 (0.00-0.23)	0.10±0.17 (0.00-0.88)	0.03±0.05 (0.00-0.23)
LCA	0.008±0.010 (0.000-0.052)	0.011±0.018 (0.000-0.096)	0.009±0.013 (0.000-0.096)	0.007±0.008 (0.000-0.025)
UDCA	0.020±0.046 (0.000-0.388)	0.009±0.018 (0.000-0.092)[a]	0.019±0.044 (0.000-0.388)	0.007±0.008 (0.000-0.024)
SLCA	0.009±0.013 (0.000-0.077)	0.022±0.028 (0.00-0.124)[a]	0.009±0.013 (0.00-0.077)	0.025±0.032 (0.00-0.124)[a]
αMCA	0.004±0.004 (0.000-0.023)	0.005±0.005 (0.000-0.022)	0.004±0.003 (0.000-0.023)	0.006±0.006 (0.000-0.022)
βMCA	0.003±0.003 (0.000-0.025)	0.004±0.003 (0.000-0.014)	0.003±0.003 (0.000-0.025)	0.004±0.004 (0.000-0.014)
HyoDCA	0.021±0.028 (0.000-0.141)	0.019±0.032 (0.001-0.154)	0.021±0.030 (0.000-0.154)	0.015±0.020 (0.001-0.079)
THCA	0.01±0.01 (0.00-0.07)	0.02±0.01 (0.00-0.04)	0.01±0.01 (0.00-0.07)	0.02±0.01 (0.00-0.04)
CA Family	53.44±83.23 (0.12-462.86)	51.59±49.37 (0.41-178.91)	50.65±80.17 (0.12-462.86)	60.15±49.11 (0.41-178.91)
CDCA Family	28.91±43.98 (0.18-161.94)	57.71±76.18 (0.49-380.70)[a]	27.56±42.30 (0.18-161.94)	75.75±85.85 (0.49-380.70)[a]
DCA Family	2.07±5.43 (0.00-39.07)	1.84±3.10 (0.00-11.67)	2.08±5.25 (0.00-39.07)	1.50±2.67 (0.00-11.67)
LCA Family	0.58±0.72 (0.01-3.04)	0.73±0.76 (0.03-3.20)	0.61±0.73 (0.00-3.04)	0.58±0.73 (0.03-3.20)
UDCA Family	0.51±2.38 (0.01-22.52)	0.37±0.44 (0.02-2.07)	0.48±2.25 (0.01-22.52)	0.45±0.49 (0.02-2.07)[a]

T-test p value (grey fill indicates significance <0.05): [a]Progressor group or Death/transplantation group is significantly different from Non-progressor or without death/transplantation outcome, respectively.

Table S6. FDR corrected p values from ANOVA.

Bile Acids	Progression FDR p values	Death Transplant Outcome FDR p values
GCA	0.14	0.10
GCDCA	0.06	0.02*
GDCA	0.77	0.38
GLCA	0.26	0.42
GUDCA	0.46	0.11
TCA	0.28	0.25
TCDCA	0.11	0.04*
TDCA	0.47	0.86
TLCA	0.14	0.56
TUDCA	0.11	0.02*
TSLCA	0.28	0.97
TMCAs	0.74	0.56
CA	0.72	0.33
CDCA	0.18	0.07
DCA	0.31	0.16
LCA	0.96	0.56
UDCA	0.11	0.33
SLCA	0.05*	0.02*
αMCA	0.98	0.93
βMCA	0.36	0.25
HyoDCA	0.96	0.95
THCA	0.22	0.38
Total BAs	0.14	0.07
Primary BAs	0.13	0.07
Secondary BAs	0.67	0.86
Glycoconjugated BAs	0.12	0.05*
Tauroconjugated BAs	0.14	0.09
Unconjugated BAs	0.64	0.56
Conjugated BAs	0.13	0.07
CA Family	0.19	0.18
CDCA Family	0.06	0.02*
DCA Family	0.95	0.83
LCA Family	0.18	0.86
UDCA Family	0.37	0.08
Ratio (CA+DCA)/(CDCA+LCA)	0.06	0.02*
Ratio CA/DCA	0.29	0.15
Ratio CDCA/LCA	0.18	0.02*
Ratio Glyco/Tauroconjugated	0.96	0.88
Ratio Primary/Secondary	0.17	0.03*
Ratio 12αOH/non/12αOH BAs	0.06	0.02*

*significance <0.05

Table S7. Univariate analysis to identify biomarkers with performance above AUC 0.7 for death/transplantation outcome in DILI patients.

Marker	AUC	95% CI	Specificity	Sensitivity	Threshold
MELD	0.88	0.77-0.98	0.67	0.99	24.56
TUDCA	0.75	0.64-0.85	0.84	0.63	0.03
GCDCA	0.73	0.6-0.86	0.79	0.65	10.95
CDCA Family	0.73	0.59-0.86	0.68	0.78	40.28
Ratio (CA+DCA)/(CDCA+LCA)	0.73	0.58-0.88	0.63	0.82	0.81
Ratio 12αOH/non-12αOH BAs	0.72	0.57-0.87	0.63	0.81	0.78
TCDCA	0.71	0.58-0.84	0.74	0.74	13.15
SLCA	0.71	0.57-0.84	0.84	0.51	0.0046
UDCA Family	0.71	0.59-0.82	0.79	0.62	0.18
Ratio CDCA/LCA	0.71	0.56-0.86	0.58	0.89	182.89
Ratio Primary/Secondary BAs	0.70	0.56-0.84	0.58	0.79	95.06

MELD, model for end-stage liver disease.

Table S8. Univariate analysis to identify biomarkers with performance above AUC 0.7 for disease progression in DILI patients.

Marker	AUC	95% CI	Specificity	Sensitivity	Threshold
MELD	0.76	0.65-0.87	0.44	0.99	24.56
SLCA	0.70	0.59-0.82	0.61	0.79	0.01
Ratio (CA+DCA)/(CDCA+LCA)	0.70	0.57-0.82	0.57	0.86	0.81

MELD, model for end-stage liver disease; SLCA, Sulfolithocholic acid; CA, cholic acid; DCA, deoxycholic acid; CDCA, Chenodeoxycholic acid; LCA, Lithocholic acid.

Table S9. Bivariate analysis to identify bile acid biomarkers which when combined with MELD have performance above AUC 0.75 for disease progression in DILI patients (n=114).

Biomarkers	AUC	Residual Deviance	IDI (95% CI)
MELD + Ratio (CA+DCA)/(CDCA+LCA)	0.82	90.92	0.137 (0.042-0.244)
MELD + Ratio 12αOH/non-12αOH BAs	0.81	91.62	0.131 (0.024-0.242)
MELD + Ratio CA/DCA	0.80	95.32	0.091 (0.009-0.199)
MELD + TCA	0.80	95.47	0.095 (0.013-0.21)
MELD + SLCA	0.81	96.09	0.077 (0.004-0.241)
MELD + Ratio Primary/Secondary BAs	0.80	97.11	0.072 (0.015-0.179)
MELD + CDCA	0.78	98.53	0.061 (0.009-0.175)
MELD + DCA	0.78	100.01	0.048 (0.001-0.125)
MELD + CA Family	0.77	98.74	0.065 (0-0.165)
MELD + Tauroconjugated BAs	0.77	100.02	0.054 (0.002-0.146)
MELD + LCA	0.77	100.59	0.04 (0-0.15)
MELD + Ratio CDCA/LCA	0.77	103.13	0.012 (-0.001-0.047)
MELD + LCA Family	0.77	104.12	0.001 (-0.002-0.031)
MELD + Secondary BAs	0.77	104.20	0.002 (-0.001-0.05)
MELD + TSLCA	0.77	104.45	-0.001 (-0.001-0.026)
MELD + GCA	0.76	100.38	0.049 (0.005-0.119)
MELD + Primary BAs	0.76	101.29	0.039 (-0.001-0.096)
MELD + Conjugated BAs	0.76	101.30	0.04 (-0.001-0.113)
MELD + Total BAs	0.76	101.46	0.038 (-0.001-0.112)
MELD + βMCA	0.76	102.15	0.023 (0-0.076)
MELD + Ratio Glyco/Tauroconjugated BAs	0.76	102.23	0.027 (0-0.115)
MELD + TCDCA	0.76	102.37	0.028 (-0.001-0.093)
MELD + Glycoconjugated BAs	0.76	102.50	0.026 (-0.001-0.077)
MELD + DCA Family	0.76	103.75	0.008 (0-0.032)
MELD + GDCA	0.76	104.05	0.006 (0-0.07)
MELD + Unconjugated BAs	0.76	104.41	0.003 (0-0.038)
MELD + UDCA	0.76	104.46	0.001 (-0.001-0.039)
MELD + αMCA	0.76	104.47	0.001 (-0.001-0.038)
MELD + HyoDCA	0.76	104.48	0.003 (0-0.049)
MELD + CA	0.76	104.50	0.003 (0-0.035)
MELD + TDCA	0.76	104.59	0 (-0.001-0.033)
MELD + GUDCA	0.76	104.68	0 (0-0.057)
MELD + UDCA Family	0.76	104.68	0 (0-0.022)

IDI, integrated discrimination improvement.

Table S10. Bivariate analysis to identify best performing bile acid biomarkers which when combined with MELD have performance ≥ AUC 0.88 for death/transplantation outcomes in DILI patients (n=116).

Biomarker pair	AUC	Residual Deviance	IDI (95% CI)
MELD + Ratio (CA+DCA)/(CDCA+LCA)	0.92	52.16	0.155 (0.009-0.28)
MELD + Ratio 12αOH/non-12αOH BAs	0.92	52.49	0.15 (0.005-0.398)
MELD + TCA	0.92	53.21	0.143 (0.011-0.272)
MELD + CA Family	0.91	55.19	0.122 (0.02-0.205)
MELD + Ratio CA/DCA	0.91	56.43	0.099 (0.002-0.179)
MELD + GCA	0.90	57.73	0.095 (0.024-0.166)
MELD + SLCA	0.90	58.67	0.062 (0.004-0.167)
MELD + Ratio Primary/Secondary BAs	0.90	59.53	0.064 (-0.001-0.187)
MELD + CDCA	0.89	58.88	0.072 (0.006-0.174)
MELD + Tauroconjugated BAs	0.89	59.02	0.081 (0-0.147)
MELD + Primary BAs	0.89	59.49	0.073 (-0.001-0.143)
MELD + THCA	0.89	61.56	0.041 (-0.001-0.21)
MELD + αMCA	0.89	62.73	0.01 (-0.001-0.093)
MELD + Conjugated BAs	0.88	60.31	0.064 (0-0.116)
MELD + Total BAs	0.88	60.32	0.063 (0-0.161)
MELD + βMCA	0.88	60.69	0.045 (0-0.134)
MELD + TCDCA	0.88	61.40	0.048 (-0.001-0.135)
MELD + CA	0.88	62.27	0.024 (0-0.067)
MELD + Ratio Glyco/Tauroconjugated BAs	0.88	62.16	0.032 (0-0.117)
MELD + LCA	0.88	63.09	0.019 (-0.001-0.071)
MELD + GLCA	0.88	63.98	0.005 (0-0.079)
MELD + GUDCA	0.88	64.00	0.002 (-0.001-0.036)
MELD + Ratio CDCA/LCA	0.88	64.01	0.003 (-0.001-0.057)
MELD + GCDCA	0.88	64.05	0.006 (-0.002-0.041)
MELD + UDCA Family	0.88	64.07	0.001 (0-0.047)
MELD + GDCA	0.88	64.13	0.001 (-0.001-0.066)
MELD + DCA Family	0.88	64.17	0.001 (-0.001-0.026)
MELD + HyoDCA	0.88	64.23	0.001 (0-0.027)
MELD + TDCA	0.88	64.23	0.001 (-0.001-0.049)
MELD + Secondary BAs	0.88	64.28	0.001 (-0.001-0.02)
MELD + Unconjugated BAs	0.88	64.29	0 (-0.001-0.025)
MELD + TUDCA	0.88	64.29	0 (-0.001-0.022)

IDI, integrated discrimination improvement; MELD, model for end-stage liver disease

Supplemental Figures

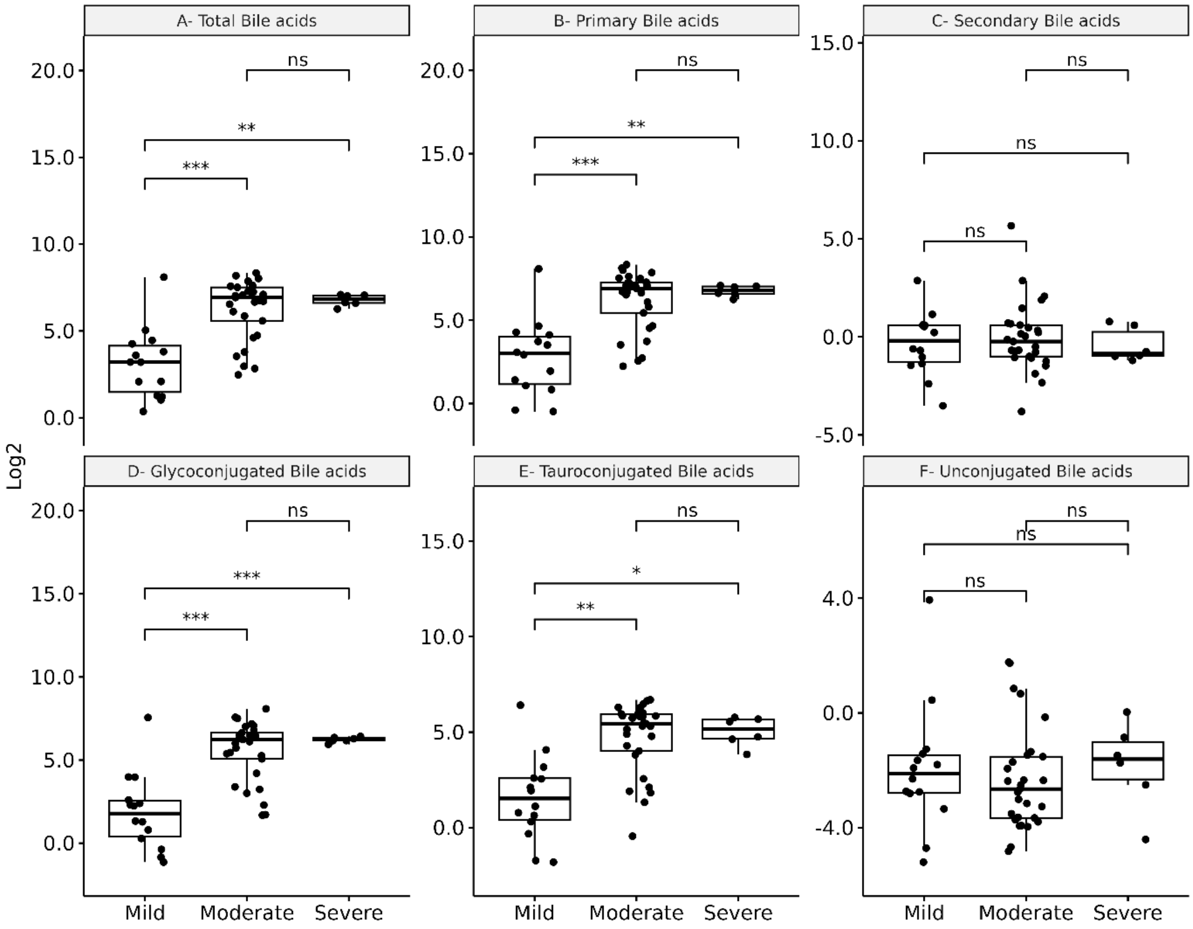

Figure S1. Levels of selected bile acids in patients with acute nonDILI stratified according to liver injury severity. Log value of µM concentration is displayed. A, Total bile acids; B, Primary bile acids; C, Secondary bile acids; D, Glycoconjugated bile acids; E, Tauroconjugated bile acids; F, Unconjugated bile acids. p values (ANOVA) adjusted by Tukey method: *** ≤ 0.001; ** ≤ 0.005; *≤0.05; ns, not significant.

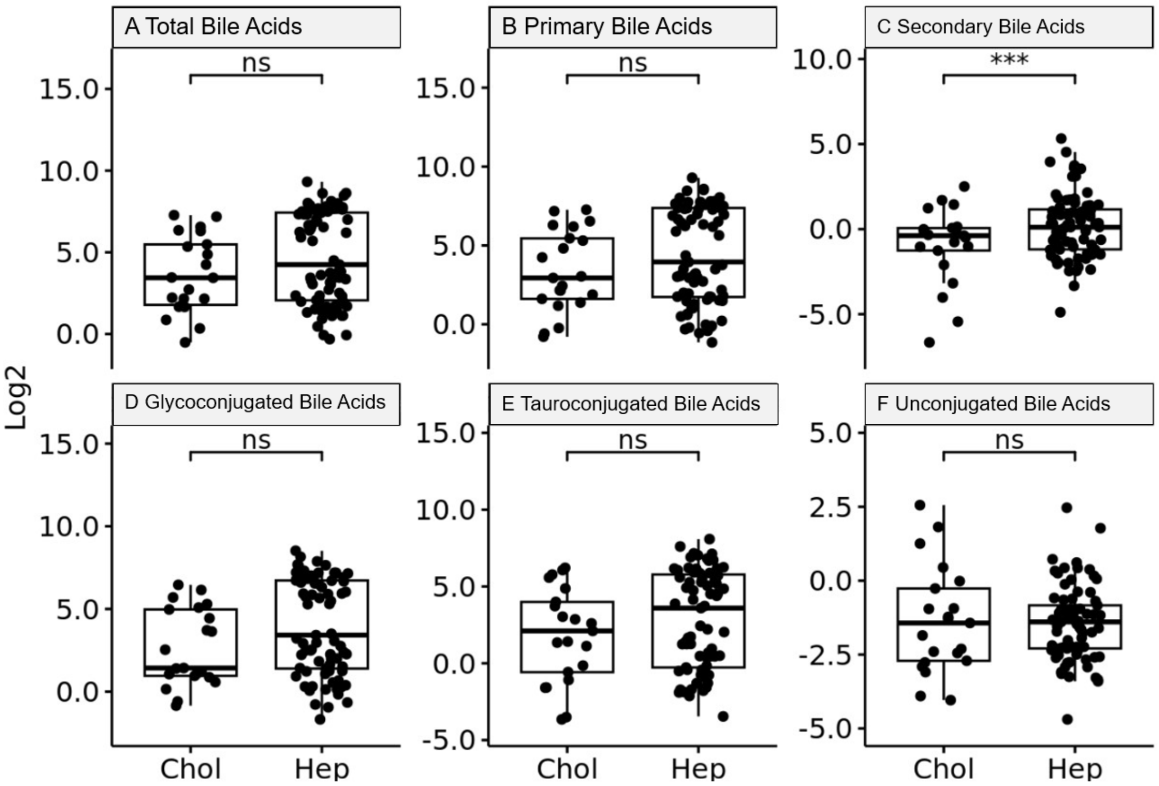

Figure S2. Levels of selected bile acids in patients with acute DILI stratified according to liver injury type. Log value of µM concentration is displayed. A, Total bile acids; B, Primary bile acids; C, Secondary bile acids; D, Glycoconjugated bile acids; E, Tauroconjugated bile acids; F, Unconjugated bile acids. T-test p values (ANOVA). Chol, Cholestatic (n=21); Hep, Hepatocellular (n=77); (Mixed injury type (n=22) is not shown); *** ≤ 0.001; ** ≤ 0.005; *≤0.05; ns, not significant.

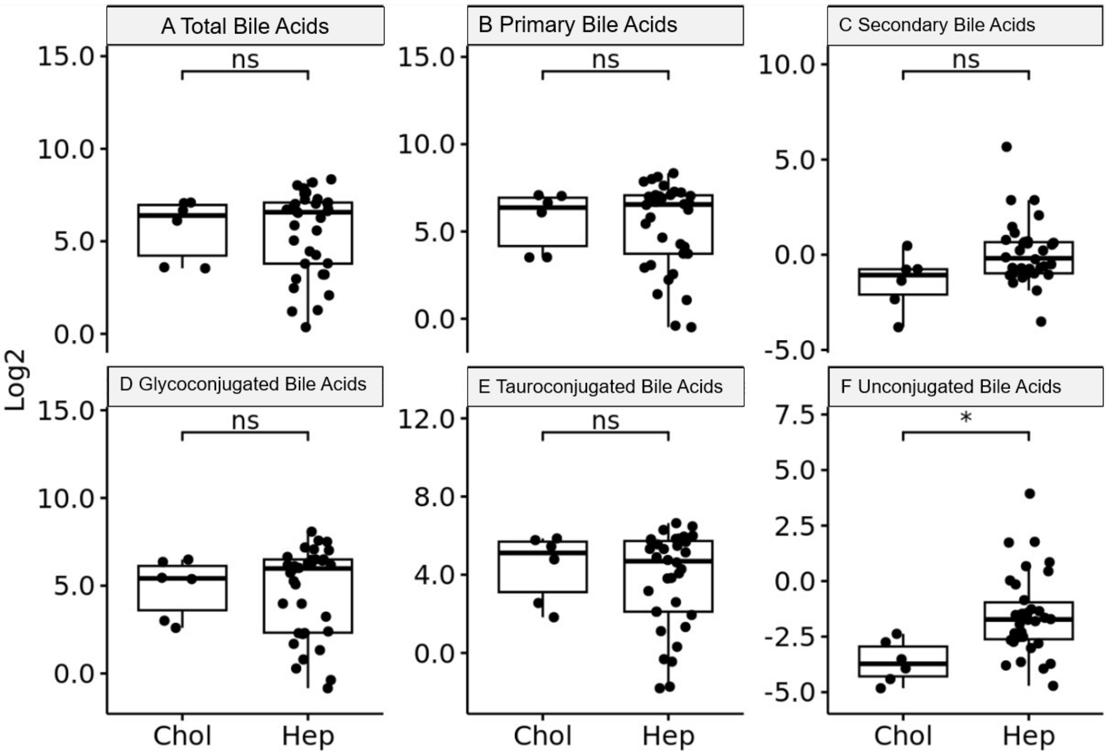

Figure S3. Levels of selected bile acids in patients with acute nonDILI stratified according to liver injury type. Log value of µM concentration is displayed. A, Total bile acids; B, Primary bile acids; C, Secondary bile acids; D, Glycoconjugated bile acids; E, Tauroconjugated bile acids; F, Unconjugated bile acids. T-test p values (ANOVA). Chol, Cholestatic (n=6); Hep, Hepatocellular (n=34) (Mixed injury type (n=9) is not shown); *** ≤ 0.001; ** ≤ 0.005; *≤ 0.05; ns, not significant.

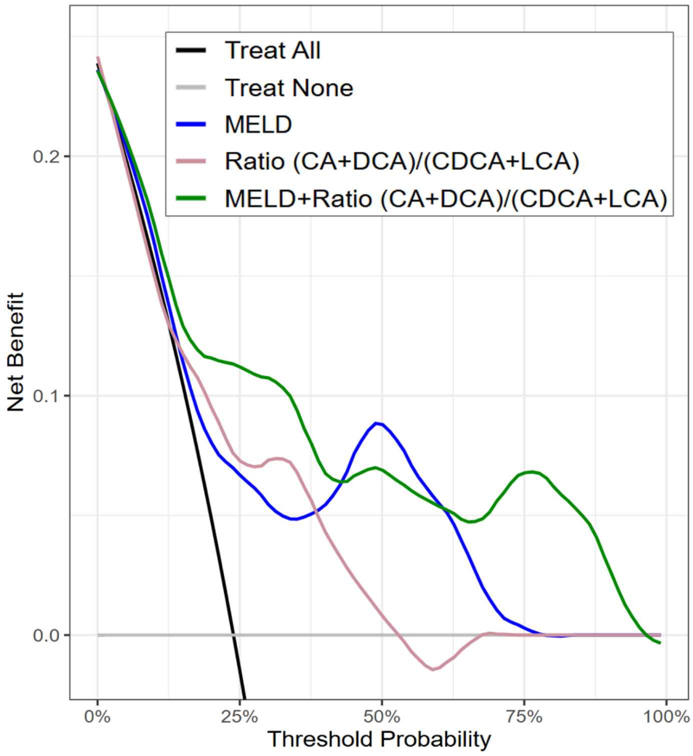

Threshold	MELD	Ratio (CA+DCA)/ (CDCA+LCA)	MELD+Ratio (CA+DCA)/ (CDCA+LCA)
0%	0.239	0.239	0.239
5%	0.199	0.201	0.204
10%	0.162	0.151	0.168
20%	0.086	0.106	0.117
30%	0.053	0.07	0.105
40%	0.05	0.035	0.062
50%	0.097	0.009	0.071

Figure S4. Decision curve analysis for progression. Net benefit of MELD and ratio of (CA+DCA)/(CDCA+LCA) is considered. CA, cholic acid; DCA deoxycholic acid; CDCA, chenodeoxycholic acid; LCA, lithocholic acid; MELD, model for end-stage liver disease.

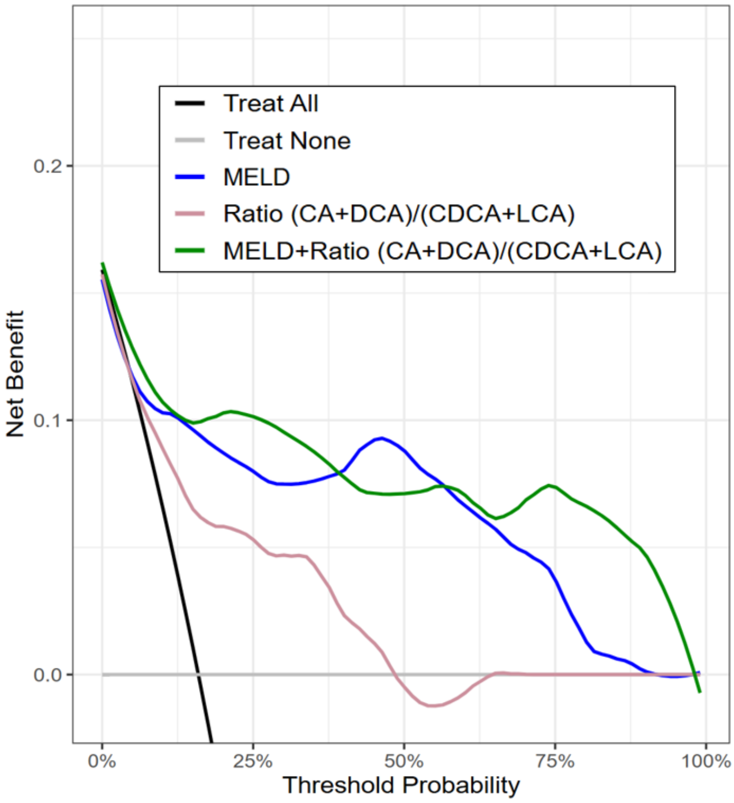

Threshold	MELD	Ratio (CA+DCA)/ (CDCA+LCA)	MELD+Ratio (CA+DCA)/ (CDCA+LCA)
0%	0.159	0.159	0.159
5%	0.113	0.108	0.126
10%	0.103	0.094	0.112
20%	0.084	0.055	0.106
30%	0.073	0.042	0.097
40%	0.083	0.015	0.074
50%	0.08	-0.009	0.071

Figure S5. Decision curve analysis for outcomes of death/liver transplantation. Net benefit of MELD and ratio of (CA+DCA)/(CDCA+LCA) is considered. CA, cholic acid; DCA deoxycholic acid; CDCA, chenodeoxycholic acid; LCA, lithocholic acid; MELD model for end-stage liver disease.

Supplemental References

1. Grove JI, Stephens C, Lucena MI, et al. Study design for development of novel safety biomarkers of drug-induced liver injury by the translational safety biomarker pipeline (TransBioLine) consortium: a study protocol for a nested case–control study. *Diagnostic and Prognostic Research* 2023; **7**(1): 18.

2. Hernanz A, Codoceo R. An improved high-performance liquid-chromatographic determination of conjugated bile acids in serum using paired-ion chromatography. *Clinica Chimica Acta; International Journal of Clinical Chemistry* 1985; **145**(2): 197-203.

3. Ye L, Liu S, Wang M, Shao Y, Ding M. High-performance liquid chromatography-tandem mass spectrometry for the analysis of bile acid profiles in serum of women with intrahepatic cholestasis of pregnancy. *Journal of Chromatography B, Analytical Technologies in the Biomedical and Life Sciences* 2007; **860**(1): 10-7.

4. Kamath PS, Kim RW. The model for end-stage liver disease (MELD). *Hepatology* 2007; **45**(3).

5. Hanley JA, McNeil BJ. The meaning and use of the area under a receiver operating characteristic (ROC) curve. *Radiology* 1982; **143**(1): 29-36.

6. Pencina MJ, D'Agostino RB, Sr., D'Agostino RB, Jr., Vasan RS. Evaluating the added predictive ability of a new marker: from area under the ROC curve to reclassification and beyond. *Statistics in Medicine* 2008; **27**(2): 157-72.

7. Pickering JW, Endre ZH. New metrics for assessing diagnostic potential of candidate biomarkers. *Clinical Journal of the American Society of Nephrology* 2012; **7**(8): 1355-64.

8. Vickers AJ, van Calster B, Steyerberg EW. A simple, step-by-step guide to interpreting decision curve analysis. *Diagnostic and Prognostic Research* 2019; **3**: 18.

9. Aithal GP, Watkins PB, Andrade RJ, et al. Case definition and phenotype standardization in drug-induced liver injury. *Clinical Pharmacology and Therapeutics* 2011; **89**(6): 806-15.
